# Drug-based mobilisation of mesenchymal stem/stromal cells improves cardiac function post myocardial infarction

**DOI:** 10.1242/dmm.049630

**Published:** 2022-11-04

**Authors:** Veneta B. Todorova, Nicoleta Baxan, Matthew Delahaye, Sian E. Harding, Sara M. Rankin

**Affiliations:** Imperial College London, Faculty of Medicine, National Heart and Lung Institute, Myocardial Function, 72 Du Cane Road, London W12 0NN, UK

**Keywords:** MSCs, Mobilisation, Drug-based mobilisation, Cardiac repair, Mirabegron, AMD3100

## Abstract

There is an unmet need for treatments that prevent the progressive cardiac dysfunction following myocardial infarction. Mesenchymal stem/stromal cells (MSCs) are under investigation for cardiac repair; however, culture expansion prior to transplantation is hindering their homing and reparative abilities. Pharmacological mobilisation could be an alternative to MSC transplantation. Here, we report that endogenous MSCs mobilise into the circulation at day 5 post myocardial infarction in male Lewis rats. This mobilisation can be significantly increased by using a combination of the FDA-approved drugs mirabegron (β_3_-adrenoceptor agonist) and AMD3100 (CXCR4 antagonist). Blinded cardiac magnetic resonance imaging analysis showed the treated group to have increased left ventricular ejection fraction and decreased end systolic volume at 5 weeks post myocardial infarction. The mobilised group had a significant decrease in plasma IL-6 and TNF-α levels, a decrease in interstitial fibrosis, and an increase in the border zone blood vessel density. Conditioned medium from blood-derived MSCs supported angiogenesis *in vitro*, as shown by tube formation and wound healing assays. Our data suggest a novel pharmacological strategy that enhances myocardial infarction-induced MSC mobilisation and improves cardiac function after myocardial infarction.

## Teaser

**Summary:** Evaluation of a drug-based mobilisation approach as a potential alternative to transplantation-based cell therapy by using mesenchymal stem/stromal cells for cardiac repair.

## INTRODUCTION

Mesenchymal stem/stromal cell (MSC) transplantation has been investigated in numerous pre-clinical and clinical trials as a method of tissue regeneration for a range of different diseases, including ischemic cardiomyopathy (Trounson and McDonald, 2015; Tompkins et al., 2018; Razeghian-Jahromi et al., 2021). Their therapeutic effects are thought to be mediated primarily by paracrine and trophic signalling, and include immunomodulation, extracellular matrix (ECM) remodelling, angiogenesis, cardiomyocyte protection, stimulation of cardiac progenitor cell proliferation and inhibition of fibrosis (Pittenger and Martin, 2004; Karantalis and Hare, 2015; Hatzistergos et al., 2010; Bagno et al., 2018; Guo et al., 2020). However, there are concerns in the field about the failure of clinical trials to replicate the promising preclinical results (Galipeau and Sensébé, 2018). An explanation for this discrepancy may be the use of allogeneic cells in human trials, which are expanded to their replicative limit and cryopreserved prior to transplantation. This is in contrast to preclinical trials, where cells are usually syngeneic, harvested during their exponential growth phase and administered fresh (Galipeau and Sensébé, 2018). Importantly, extensive culture expansion can cause these cells to become senescent, which affects their gene expression, limits their homing abilities and interferes with their localisation to the site of injury (Honczarenko et al., 2006; Rombouts and Ploemacher, 2003).

To address these issues, we propose a novel approach of harnessing the therapeutic potential of endogenous MSCs, by increasing their mobilisation into the blood by using drugs. As a natural response to injury, such as skeletal trauma, skin burns, cardiomyopathies and acute myocardial infarction, stem/progenitor cells (including MSCs) mobilise into the circulation (Shintani et al., 2001; Wojakowski et al., 2004; Massa et al., 2005; Caplan and Correa, 2011; Girousse et al., 2021; Kucia et al., 2004). This is considered to be a part of the wound healing response, and we propose that, by increasing the extent of MSC mobilisation following injury through drugs, we can accelerate the resolution of inflammation and enhance tissue regeneration. Indeed, this strategy has already been validated in the context of skin wound and bone fracture healing (Hong and Son, 2014; Meeson et al., 2019). Such a drug-based regenerative approach would circumvent the cost and technical challenges associated with harvesting, culture expansion and transplantation of MSCs. Our goal here was to validate a pharmacological strategy to increase MSC mobilisation post myocardial infarction, and to test whether it can improve cardiac repair and function at the chronic stage (5 weeks) after myocardial infarction.

It is important to distinguish this work from previous research, which has investigated the use of granulocyte colony-stimulating factor (G-CSF) therapy for cardiac regeneration. Indeed, three double-blind placebo-controlled clinical trials showed that haematopoietic stem/progenitor cell (HSPC) mobilisation using G-CSF is ineffective at restoring the left ventricular ejection fraction (LVEF) (Zohlnhöfer, 2008; Engelmann et al., 2006; Ripa et al., 2005). These findings suggested that G-CSF treatment does not mobilise pro-reparative cell types, and it was later reasoned that MSCs would be a better cell type for regeneration (Ripa et al., 2006). We have previously reported that G-CSF alone or in combination with the CXCR4 antagonist AMD3100 does not mobilise MSCs, whereas the β3-AR agonist BRL37344 in combination with AMD3100 (Plerixafor) mobilises both MSCs and HSPCs (Pitchford et al., 2009; Fellous et al., 2020), thus having greater potential as a pro-reparative therapy. In support of this, in a model of bone healing we showed that the BRL37344+AMD3100 drug combination enhances tissue repair (Fellous et al., 2020).

In this study, we chose to use the β3 adrenergic receptor (β3AR) agonist mirabegron as it has been approved by the United States Food and Drug Administration (FDA) and is thus more suitable for potential future translation into the clinic. Mirabegron has been approved for the treatment of overactive bladder disease and is also currently in a phase III clinical trial for heart failure (Warren et al., 2016; Pouleur et al., 2018). AMD3100 is FDA approved for use in combination with G-CSF for mobilisation of HSPCs to peripheral blood for collection and subsequent transplantation in patients with non-Hodgkin lymphoma or multiple myeloma (Wang et al., 2020), and was extensively studied in stem/progenitor cell mobilisation (Pitchford et al., 2009; Fellous et al., 2020; Redpath et al., 2017; Dar et al., 2011; Xu et al., 2011). Using a Lewis rat myocardial infarction model, we first identified the optimal time for pharmacological mobilisation by determining the peak day of myocardial infarction-induced MSC egress into the circulation. We then tested the ability the mirabegron and AMD3100, alone or in sequential combination, to enhance this myocardial infarction-induced mobilisation at day 5 post myocardial infarction and showed that only combination therapy, i.e. sequential treatment with mirabegron+AMD3100 (MA treatment) was successful. The effect of the MA treatment on cardiac function at 5 weeks post myocardial infarction was then evaluated.

## RESULTS

### Myocardial infarction mobilises endogenous stem/progenitor cells into the circulation

**Fig. 1.**
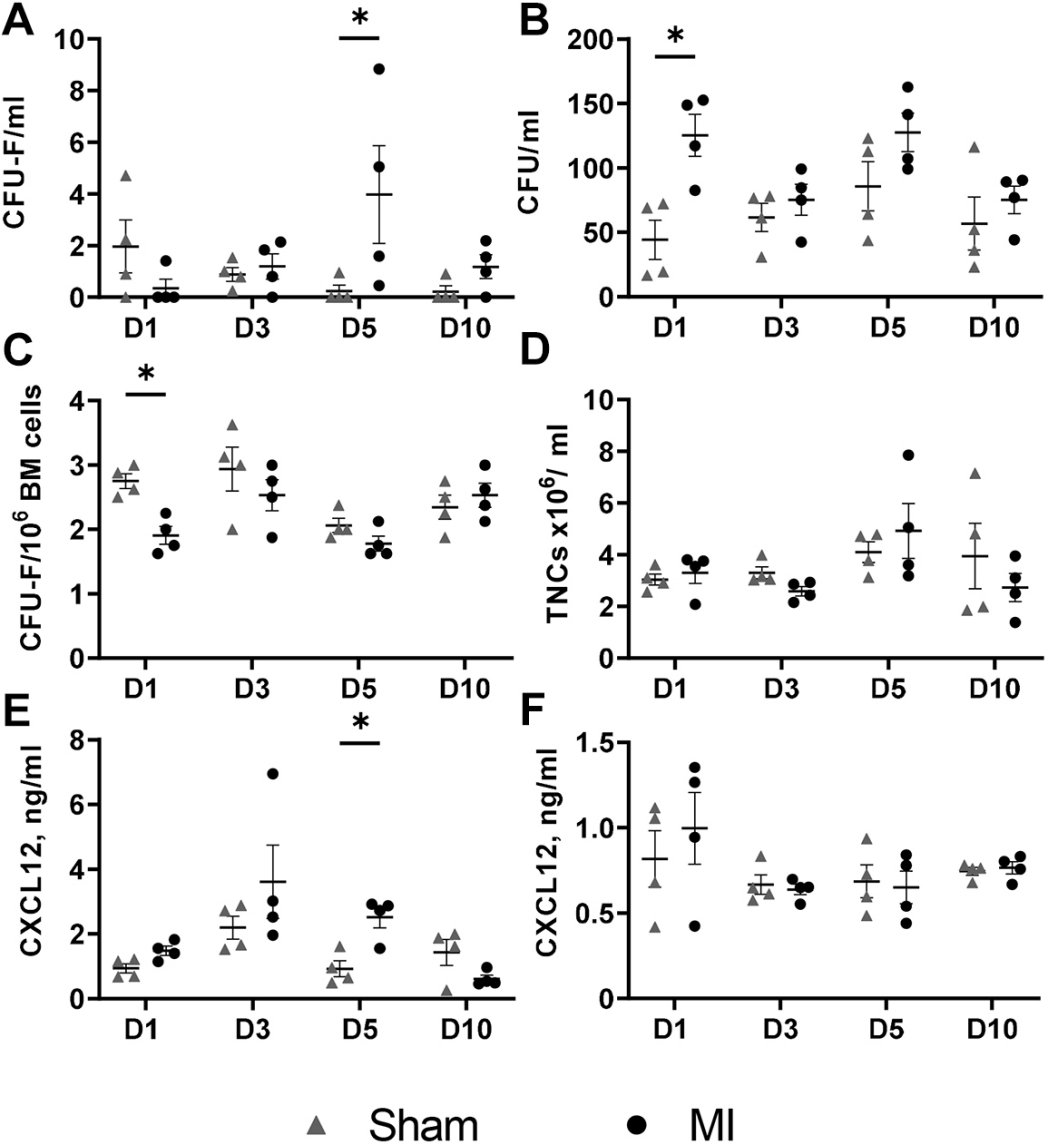
**Dynamics of stem/progenitor cell mobilisation after myocardial infarction.** Male Lewis rats were sacrificed on day (D)1 to D10 after myocardial infarction (MI) (*n*=4 for each timepoint), or sham (*n*=4 for each timepoint) surgery, and their blood and bone marrow (BM) were collected for analysis. (A-C) To analyse endogenous myocardial infarction-induced mobilisation of stem/progenitor cells into the blood, we quantified the number of circulating MSCs (in CFU-F/ml) (A), circulating HSPCs (in CFU/ml) (B) and resident BM MSCs (in CFU-Fs per 1×10^6^ seeded BM cells, i.e. in CFU-F/million) (C). (D) Circulating nucleated cells (TNCs) were measured in whole blood by using Turk's solution and are presented as total cells×10^6^/ml. (E,F) Levels of CXCL12 in the plasma (E) or BM (F) were measured (in ng/ml) using ELISA. Two-way repeated Measures ANOVA was performed on all datasets, Šidák's multiple comparisons test was performed on datasets A-C and E (*P<0.05).

To assess the endogenous myocardial infarction-induced mobilisation of stem/progenitor cells into the blood, Lewis rats underwent either permanent ligation of the left anterior descending artery (LAD), leading to myocardial infarction or underwent control surgery (sham), whereby the suture was only passed through the tissue without tying the LAD. Four paired groups (myocardial infarction and sham, *n*=4 each, total of 32 animals for the study) were terminated at different timepoints after myocardial infarction surgery, i.e. day 1, day 3, day 5 and day 10, and the numbers of circulating HSPCs and MSCs were assessed by quantifying colony-forming units (CFUs) and colony forming unit–fibroblasts (CFU-Fs), respectively. Circulating MSCs were significantly elevated on day 5 in the myocardial infarction group compared with those in the sham group (0.24±0.47 (sham, *n*=4) vs 3.98±3.78 (myocardial infarction, *n*=4) CFU-F/ml, *P*=0.0142) (Fig. 1A), whereas HSPCs were significantly elevated on day 1 (44.27±30.4 (sham, *n*=4) vs 125.3±32.52 (myocardial infarction, *n*=4) CFU/ml, *P*=0.043) (Fig. 1B). Furthermore, a lower level of bone marrow CFU-Fs was observed in the myocardial infarction group on day 1 compared to that in the sham group (Fig. 1C). There was no difference in the total circulating nucleated cells between sham and myocardial infarction at any timepoint (Fig. 1D). The plasma and bone marrow levels of chemokine CXCL12 were quantified by using sandwich enzyme-linked immunosorbent assay (ELISA). There was no difference in the concentration of CXCL12 in bone marrow aspirates of sham and myocardial infarction groups (Fig. 1F); however, in the myocardial infarction group, plasma levels of CXCL12 were significantly increased on day 5 after injury (Fig. 1E).

### Combined sequential treatment with mirabegron+AMD3100 increased levels of circulating stem/progenitor cells after myocardial infarction

**Fig. 2.**
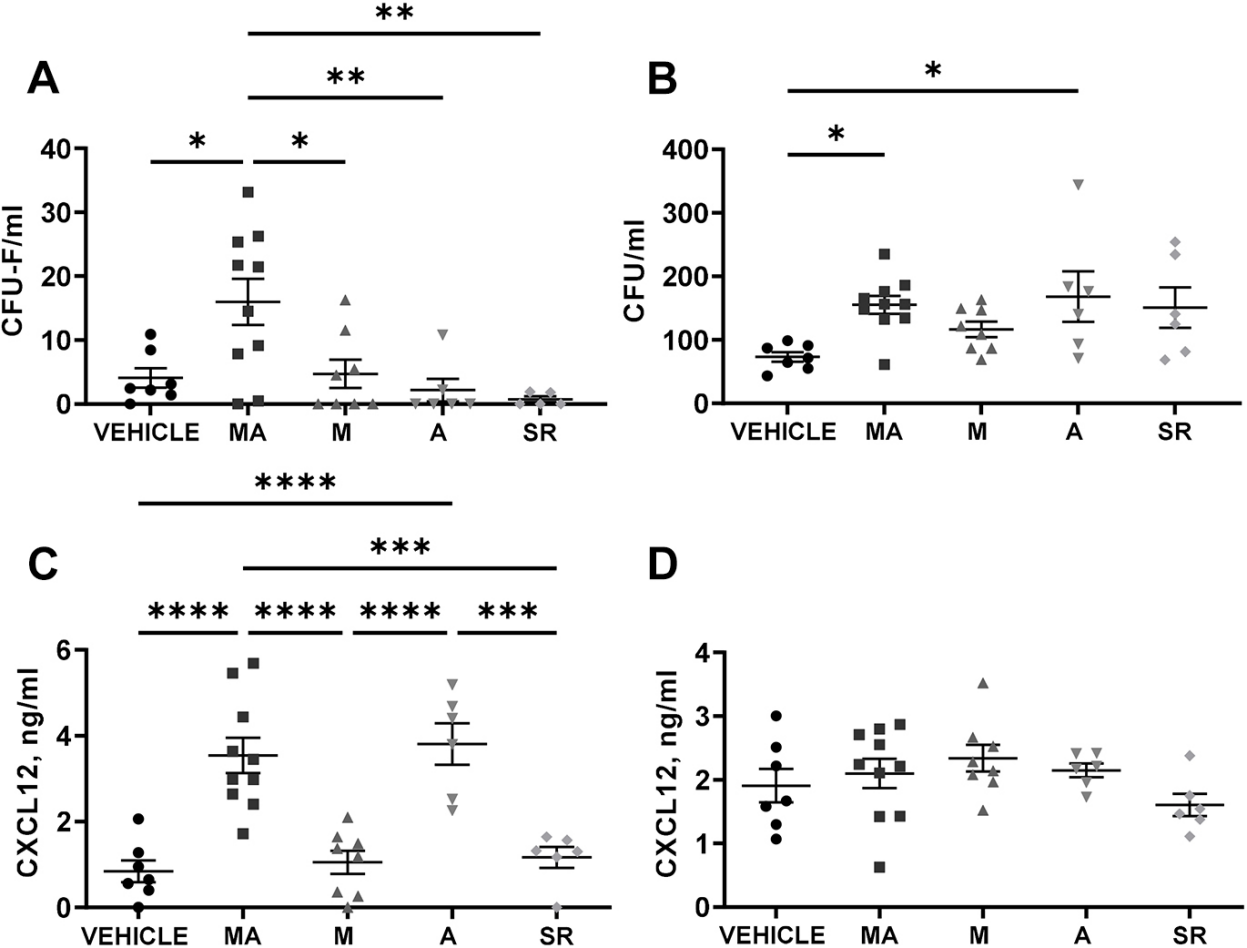
**Effect of drugs on stem/progenitor cell mobilisation after myocardial infarction.** Five different treatment groups were assessed, i.e. rats after myocardial infarction surgery treated with vehicle (*n*=7), mirabegron followed by AMD3100 (MA; *n*=10), mirabegron only (M; *n*=8), AMD3100 only (A; *n*=6) or SR59230A only (SR; *n*=6). (A) Amounts of circulating MSCs were measured as CFU-F/ml. (B) Levels of circulating HSPCs were measured as CFU/ml. (C,D) Levels of CXCL12 in plasma (C) and bone marrow (D) were quantified using ELISA, and plotted in ng/ml. One-way ANOVA was performed on all datasets, Tukey's multiple comparisons test was performed on A-C, *P<0.05, **P<0.01, ***P<0.001, ****P<0.0001.

**Fig. 3.**
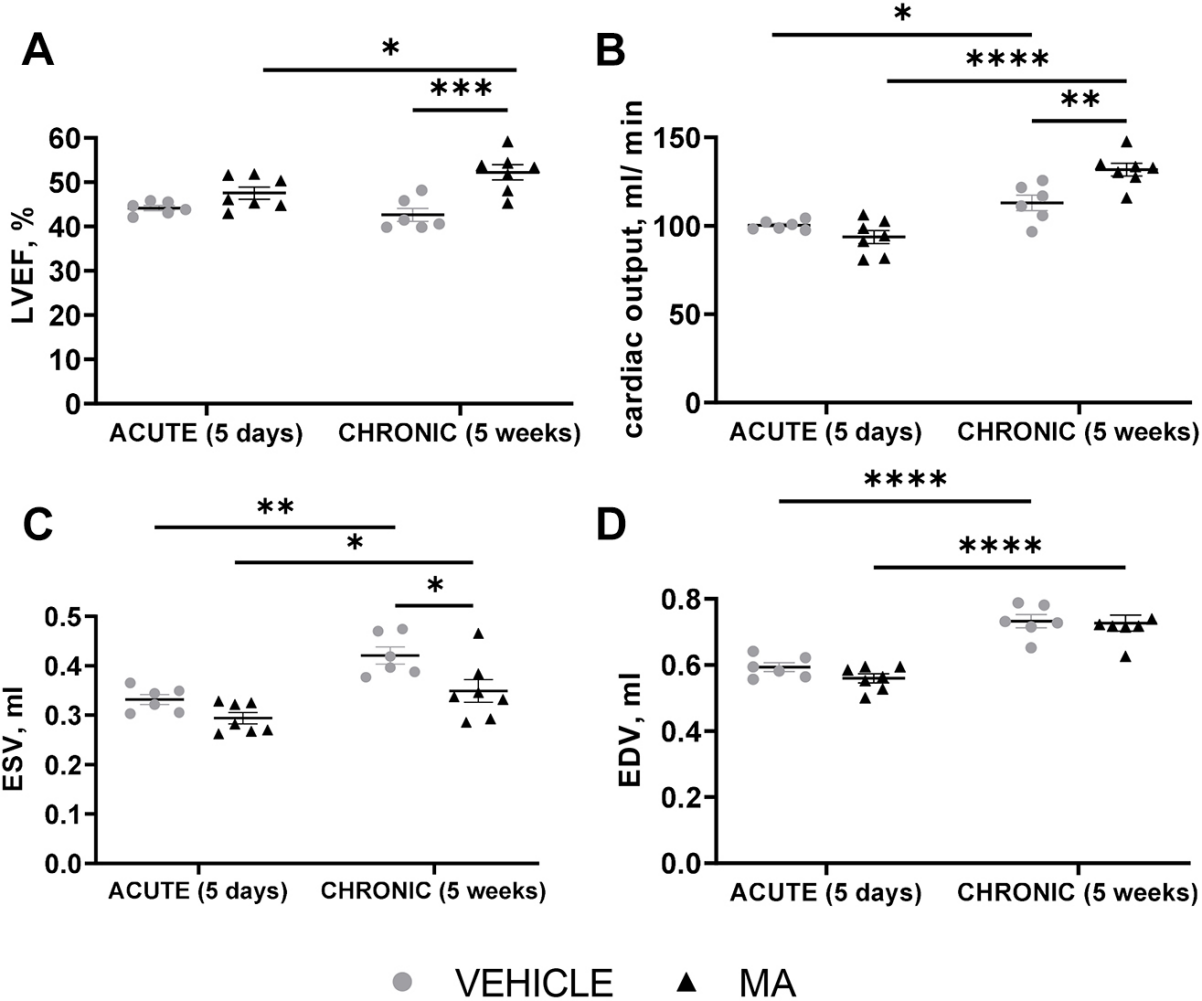
**MA-treated rats showed improved global cardiac contractility at 5 weeks post myocardial infarction compared to vehicle-treated rats.** Male Lewis rats underwent myocardial infarction surgery followed by treatment with vehicle (*n*=6) or combined sequential treatment with mirabegron+AMD3100 (MA; *n*=7). (A-D) Cardiac function was validated by measuring left ventricular ejection fraction (LVEF) (in percentage) (A), cardiac output (in ml/min) (B), end-systolic volume (ESV) (in ml) (C) and end-diastolic volume (EDV) (in ml) (D) after treatment. Measurements were taken at day 5 and week 5 post myocardial infarction surgery, using blinded cardiac MRI. Two-way ANOVA was performed on all datasets, with Bonferroni post-tests on datasets A-C (*P<0.05, **P<0.01, ***P<0.001) and Šidák's multiple comparisons test on datasets A-D (*P<0.05, **P<0.01, ****P<0.0001).

After establishing the peak day (day 5) of myocardial infarction-induced endogenous MSC mobilisation into the circulation, we investigated whether this can be further increased by application of mirabegron (daily gavage over 5 days) or AMD3100 (once at day 5) alone (M or A group, respectively) (Fig. S2B,C), or in combination (MA group) (Fig. S2A). In addition, we tested whether treatment with the β_3_AR antagonist SR59230A affects myocardial infarction-induced MSC mobilisation. For this, all animal groups underwent myocardial infarction surgery and, 24 h later, were given a daily dose of mirabegron (M), SR59230A (SR) or vehicle only for 5 days (Fig. S2C-E). The MA group was treated with mirabegron for 5 days, but, on the last day – 1 h after the last dose of mirabegron, animals also received a single dose of AMD3100 (Fig. S2A). The AMD3100 only (A) group received no pre-treatment but a single dose of AMD3100 on day 5 (Fig. S2B). Animals were sacrificed 1 h after the last drug had been administered. The results showed that mirabegron (M) or AMD3100 (A) alone did not significantly increase circulating levels of CFU-Fs; however, sequential administration of both drugs (MA) caused an ∼4-fold increase compared to administration with vehicle (vehicle: 4.075±4.02, *n*=8 vs MA: 15.99±11.36 CFU-F/ml, *n*=10, *P*<0.05) (Fig. 2A). When using the same treatment protocols, these drugs did not induce MSC mobilisation in rats that had not undergone myocardial infarction surgery (MA, *n*=4, M, *n*=4, A, *n*=4, V, *n*=4). Both groups that had received AMD3100 showed elevated levels of circulating HSPCs and increased of CXCL12 plasma levels (Fig. 2B,C). No difference was seen in the bone marrow CXCL12 levels after any of the drug treatments (Fig. 3D). Whereas treatment with SR59230A led to the lowest levels of circulating CFU-Fs (Fig. 2A), no significant inhibition of myocardial infarction-induced MSC mobilisation was observed.

The cells that we here refer to as MSCs grew from the blood and bone marrow samples as CFU-Fs, and were plastic adherent with fibroblast-like appearance under a light microscope (Fig. S6A). Following their expansion in culture, they were analysed by using flow cytometry for their expression of MSC-like cell surface markers. The bone marrow-derived cells had a significantly larger CD45-positive population compared to their blood-derived counterparts [39.47±6.4%, bone marrow (*n*=6) vs 3.89±2.8%, blood (*n*=6), *P*=0.0022] (Fig. S6E). Culture-expanded bone marrow- and blood-derived CFU-Fs (>P5) were 96.8±0.28% and 97.82±1% positive for CD90 and CD29, respectively (gated on cells negative for CD45, CD31, CD43 and His48) (Fig. S6C,D), and successfully underwent chondrogenesis and osteogenesis; however, only the bone marrow-derived CFU-Fs from the vehicle and SR groups underwent adipogenesis (Fig. S6B).

### MA treatment improved heart function at 5 weeks post myocardial infarction compared to treatment with vehicle

Data presented in Fig. 2 led us to next investigate whether MA treatment has an effect on cardiac function post myocardial infarction. To do this, we performed myocardial infarction surgery on Lewis rats and used blinded cardiac magnetic resonance imaging (MRI) analysis to investigate animals treated with vehicle (*n*=6) or MA (*n*=7) at day 5 (acute) and week 5 (chronic) after surgery. No changes in cardiac function were observed after treatment with vehicle or MA at day 5 post myocardial infarction (Fig. 3A-D). By contrast, at 5 weeks post myocardial infarction, the MA-treated group showed improved LVEF (vehicle: 42.61±3.53% vs treated: 51.99±4.43%, *P*<0.001) (Fig. 3A), cardiac output (vehicle: 112.96±10.7 ml/min vs treated: 132.03±9.58 ml/min, *P*<0.01) (Fig. 3B) and decreased end-systolic volume (ESV) (vehicle: 0.42±0.04 ml vs treated: 0.35±0.06 ml, *P*<0.05) (Fig. 3C). The end-diastolic volume (EDV) was increased between acute and chronic stages but not affected by MA treatment (Fig. 3D). Moreover, LVEF increased between day 5 and week 5 in the MA-treated but not the vehicle-treated group.

**Fig. 4.**
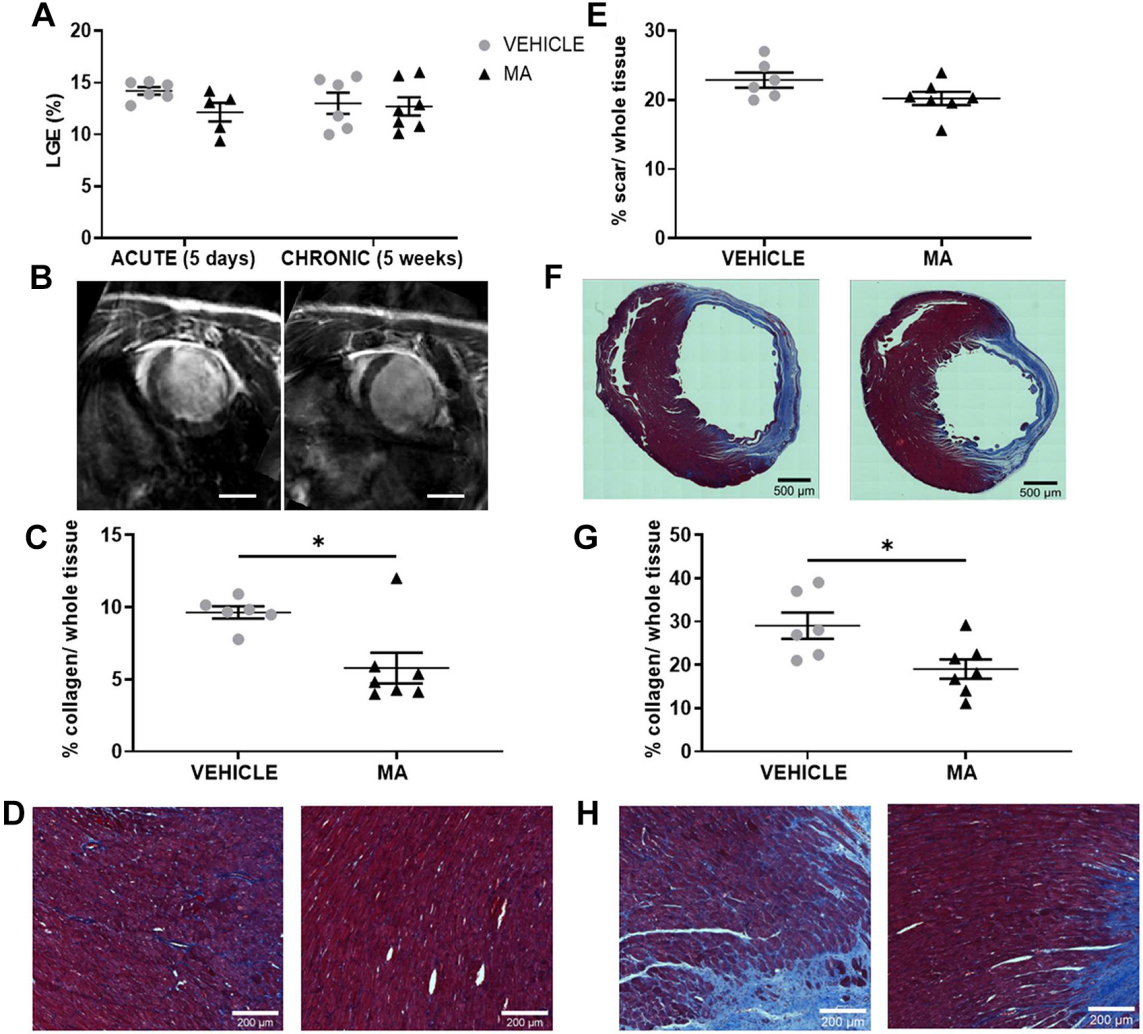
**Scar size and interstitial fibrosis.** Male Lewis rats underwent myocardial infarction surgery followed by treatment with vehicle (grey circles; *n*=6) or combined sequential treatment with mirabegron+AMD3100 (MA; black triangles; *n*=7). (A,E) The size of the myocardial scar (in %) was assessed after 5 days and 5 weeks by using the late gadolinium enhancement (LGE) method *in vivo* (two-way ANOVA, vehicle *n*=6, MA, *n*=7) (A) or after 5 weeks only by using Masson's trichrome method *ex vivo* (unpaired Student's *t*-test, *P*=0.0935, vehicle *n*=6, MA, *n*=7) (E). (B) Representative images of rat hearts from the vehicle (left) or MA (right) groups analysed using LGE (images from the 5 weeks post myocardial infarction timepoint). Scale bars: 6 mm. (F) Representative images of Masson's trichrome-stained heart sections after treatment with vehicle (left) or MA (right); collagen is shown in blue, muscle tissue is shown in red. (C,G) Masson's trichrome-stained images were also used to quantify the interstitial fibrosis at 5 weeks post myocardial infarction as collagen (in %) over the whole tissue: healthy region of the left ventricle (Mann–Whitney test, *P*=0.0350) (C), border zone of the infarct (unpaired Student's *t*-test, *P*=0.0208, vehicle, *n*=6, MA *n*=7) (G). (D,H) Representative images of interstitial fibrosis in the healthy heart region after treatment with vehicle (left) or MA (right) (D), and in the border zone after treatment with vehicle (left) or MA (right) (H).

We then measured the size of the myocardial scar using two methods. Late gadolinium enhancement (LGE) was used *in vivo* at 5 days and 5 weeks; Masson's trichrome staining of heart sections was used *ex vivo* at 5 weeks post myocardial infarction. We did not observe differences in scar size between vehicle- and MA-treated groups at any timepoint (Fig. 4A,B). By using the Masson's trichrome-stained sections, we quantified the extent of interstitial fibrosis in the healthy region of the left ventricle and the border zone of the infarct. This analysis showed reduced interstitial fibrosis (collagen in %) in both healthy myocardium and the border zone of the infarct of the MA-treated group (Fig. 4C,D).

### MA treatment increased blood vessel density in the border zone of the infarct

**Fig. 5.**
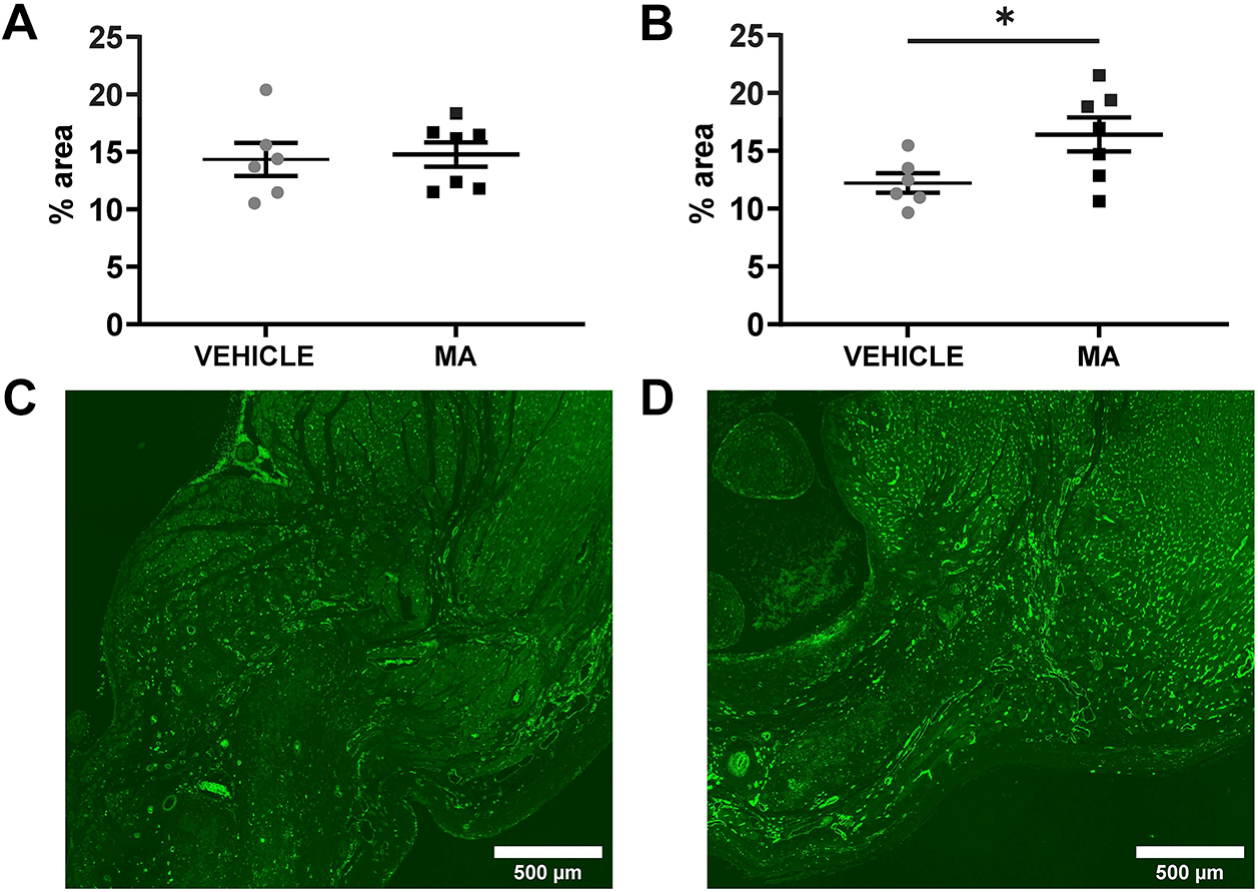
**Blood vessel density in the heart at 5 weeks post myocardial infarction.** Blood vessel density was quantified by staining with isolectin-B4 in the heart tissue of vehicle-treated (grey circles; *n*=6) and MA-treated (black squares; *n*=7) rats 5 weeks after myocardial infarction surgery. (A,B) Plotted is the isolectin-B4-positive area (in %) for the scar zone (A) and the border zone (B) (unpaired Student's *t*-tests, *P*=0.8104 and *P*=0.0376, respectively). (C,D) Representative images of isolectin-B4-stained border zone after treatment with vehicle (C) or MA (D).

To determine whether MA treatment for MSC mobilisation affects blood vessel density, heart sections obtained from rats 5 weeks after myocardial infarction surgery were stained with the blood vessel marker isolectin-B4. Our blinded analysis showed that the MA-treated group had increased blood vessel density in the border zone of the infarct (Fig. 5B). No difference in blood vessel density within the scar tissue was seen between the groups (Fig. 5A).

### Blood- and bone marrow-derived MSCs support angiogenesis *in vitro*

**Fig. 6.**
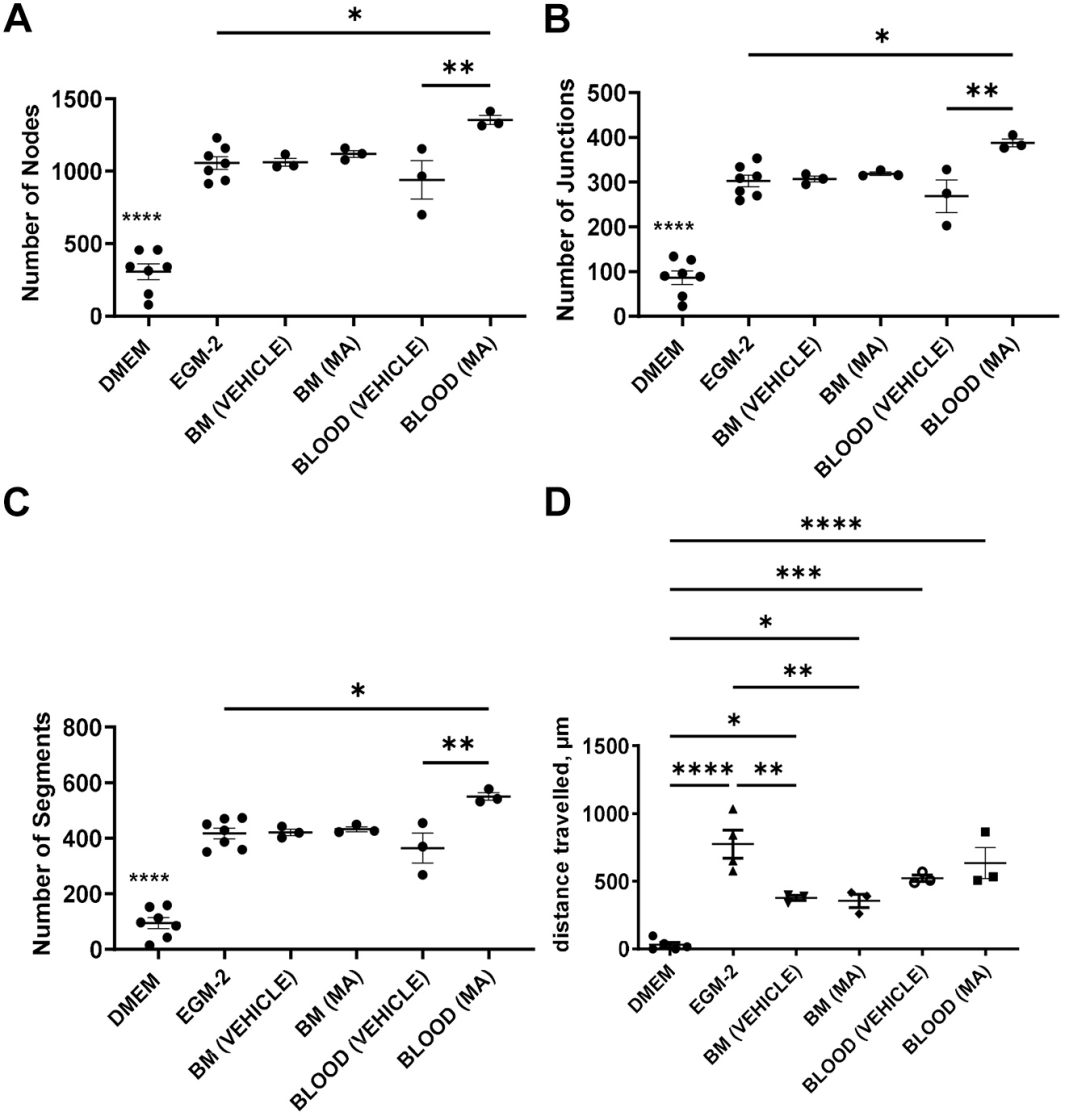
***In vitro*** **HUVEC tube formation assay to assess the ability of blood and bone marrow MSC conditioned medium to support angiogenesis.** Plotted are the effects conditioned media obtained from bone marrow- (BM) and blood-derived MSCs treated with vehicle or MA have on HUVEC tube formation. (A-D) Carried out were two *in vitro* angiogenesis assays, one assessing the ability of HUVECs to form a tube-like structure – quantified by the number of nodes (A), junctions (B) and segments (C) – and one assessing HUVEC migration in the context of wound healing (closing a manually introduced gap by scratching the plate) (D), quantified as distance travelled (µm) during 24 h. DMEM was used as negative control, EGM-2 as positive control. One-way ANOVA, Tukey's multiple comparisons test, *P<0.05, **P<0.01, ***P<0.001, ****P<0.0001, *n*=3.

We next explored whether mobilised blood-derived MSCs secreted pro-angiogenic factors. To assess this, we compared the angiogenic potential of conditioned medium from blood- and bone marrow-derived MSCs *in vitro* by using human umbilical vein endothelial cell (HUVEC) tube formation and wound healing assays. The blood and bone marrow-derived MSCs used for these assays were culture expanded (>P5) after isolation from the MA and vehicle groups (day 5 post myocardial infarction). Conditioned medium was obtained by incubating the cells with serum-free DMEM for 24 h. Complete EGM-2 and serum-free DMEM served as positive and negative controls, respectively. Our results indicate that the conditioned medium from all MSCs significantly increased HUVEC tube formation compared to negative control (DMEM) (Fig. 6A-C). However, only the conditioned medium from the blood-derived MSCs from the MA group was significantly better than EGM-2 at supporting tube formation (Fig. 6A-C). Of note, conditioned medium from the blood-derived MSCs of the MA group had higher levels of secreted CXCL12 compared to its counterpart from bone marrow-derived MSCs (Fig. S8).

In the wound healing assay, all conditioned media mediated significantly more efficient wound closure compared to the DMEM negative control (Fig. 6D). However, whereas bone marrow MSC conditioned medium performed worse than EGM-2 positive control, blood MSC conditioned medium was no different, indicating that it is as good as the positive control at supporting wound closure (Fig. 6D).

### MA treatment at 5 weeks post myocardial infarction decreases levels of IL-6 and TNF-α in plasma

**Fig. 7.**
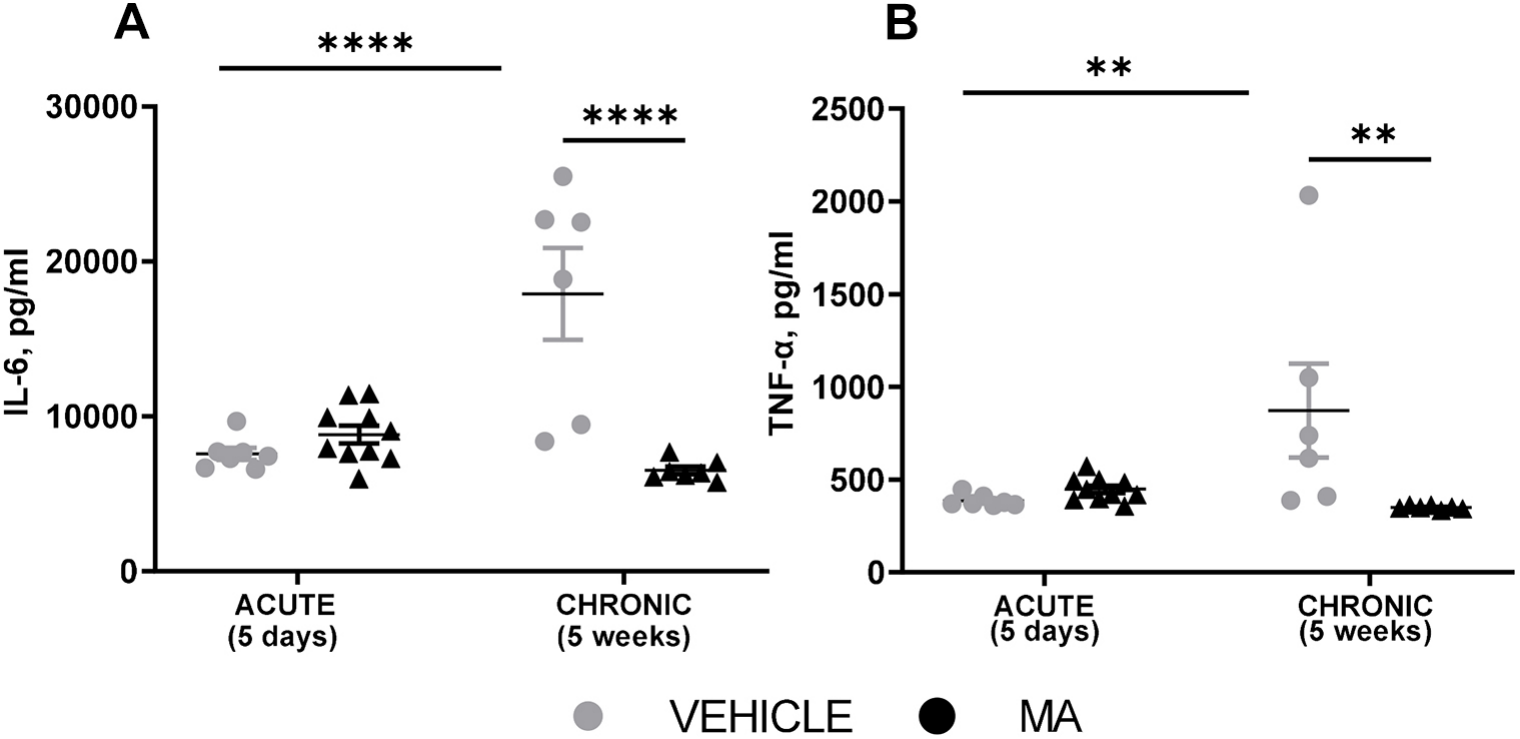
**Circulating cytokines on day 5 and week 5 post myocardial infarction.** Male Lewis rats underwent myocardial infarction surgery followed by treatment with vehicle (grey circles) or combined sequential treatment with mirabegron+AMD3100 (MA; black triangles) after 5 days (acute) or 5 weeks (chronic). Sandwich ELISA was performed on plasma samples from vehicle- and MA-treated groups to determine whether MA treatment affects the levels of the pro-inflammatory cytokines IL-6 and TNF-α. (A,B) Plotted are levels of IL-6 (A) and TNF-α (B) on day 5 (vehicle *n*=7, MA, *n*=10) and week 5 (vehicle *n*=6, MA, *n*=7) post myocardial infarction. Results are shown in pg/ml. All datasets were analysed using two-way ANOVA and Šidák's post-tests, **P<0.01, ****P<0.0001.

Another known therapeutic effect of MSCs is immunomodulation. Thus, we investigated whether increasing numbers of circulating MSC at day 5 after myocardial infarction impacted the systemic inflammatory response. Analysis of plasma cytokine levels was performed using blood samples from the drug selection study with an endpoint at day 5, and the functional improvement study with an endpoint at week 5 post myocardial infarction. The data revealed that, at week 5 post myocardial infarction, MA treatment was associated with significant reductions in circulating levels of both IL-6 and TNF-α compared to levels after treatment with vehicle (Fig. 7A,B). Plasma levels of IL-10 did not differ between the groups at any timepoint but significantly increased over time in both groups (Fig. S9). Our data also show significant elevation of both IL-6 and TNF-α from day 5 to week 5 in the vehicle group, which was not observed in the MA-treated group (Fig. 7A,B). Although numbers of circulating MSCs and TNCs did not differ between the groups at 5 weeks post myocardial infarction, numbers of circulating HSPCs were significantly reduced in the MA group compared to that treated with vehicle (Fig. S10). These data suggest that MA treatment significantly reduces some aspects of the inflammatory response at 5 weeks post myocardial infarction.

## DISCUSSION

Despite promising preclinical studies investigating culture-expanded MSC transplantation for cardiac repair, clinical trials using this approach have been largely disappointing (Galipeau and Sensébé, 2018). This may be due to technical and practical issues associated with culture expansion, handling and delivery of cell therapies. An alternative approach that circumvents these problems is to harness the regenerative potential of endogenously mobilised MSCs. In this study, we explored the use of drugs that enhance myocardial infarction-induced mobilisation of MSCs into the blood to promote cardiac tissue repair. Previous work from our group has identified a drug-based MSC mobilisation strategy (Pitchford et al., 2009; Redpath et al., 2017; Dar et al., 2011; Xu et al., 2011) that improved healing of orthopaedic injuries (Fellous et al., 2020). Here, we suggest that this regenerative pharmacology approach has potential for improving heart function post myocardial infarction.

In the first 24 h post myocardial infarction, there is an acute sterile inflammation response associated with the influx of inflammatory cells; this is followed by a resolution and repair response that commences 5-7 days post myocardial infarction, and leads to neovascularisation and scar formation (Prabhu and Frangogiannis, 2016). Our data show that, whereas myocardial infarction-induced mobilisation of HSPCs peaks at day 1, mobilisation of MSCs is delayed, peaking at day 5, coinciding with the transition to the resolution of inflammation (Ong et al., 2018). Compared to treatment with vehicle, the sequential treatment with the β3-AR agonist mirabegron and the CXCR4 antagonist AMD3100 (MA treatment) led to significant increase in circulating MSCs at day 5 post myocardial infarction. MA treatment resulted in improved left ventricular function at 5 weeks post myocardial infarction, increased LVEF (vehicle vs treated: 42.61±3.53% vs: 51.99±4.43%, respectively; *P*<0.001) and reduced ESV. The LVEF results show a trend towards an almost normal LVEF, which in rat has been estimated to be ≥51.6% (Higuchi et al., 2007), and is close to the normal level of LVEF in humans (≥50%) (Ponikowski et al., 2016). The EDV was increased at week 5 (chronic stage) compared to day 5 (acute stage) in both treatment groups, which indicates the development of a heart failure phenotype. This was not attenuated by the MA drug combination, suggesting that our treatment did not prevent dilatation but improved systolic function. The increased contractility could, at least partly, be due to reduced levels of plasma IL-6 and TNF-α in the MA-treated group, decreased interstitial fibrosis and increased angiogenesis at the border zone of the infarct. It should be noted that we exclude the possibility of direct effects of the drug on cardiac function at the 5-week timepoint because both drugs would have been completely cleared by then.

Previous studies have shown that IL-6 and TNF-α can both act as a cardiodepressant (Odeh, 1993; Pathan et al., 2011); thus, by lowering their levels we might have alleviated some of their cardiodepressive effects. Plasma levels of IL-6 increase with the severity of chronic heart failure and can – independently of LVEF levels – be prognostic for heart failure patients, showing the importance of this cytokine in the pathophysiology of the disease (Tsutamoto et al., 1998; Mann, 2015). IL-6 reduces the peak systolic Ca^2+^ transient and contractility by stimulating nitric oxide (NO) production, followed by a subsequent cyclic GMP-mediated dampening of L-type Ca^2+^ channel currents. IL-6 has also been shown to achieve sustained cardiodepression by directly triggering expression of inducible nitric oxide synthase (iNOS) in isolated cardiomyocytes (Finkel et al., 1992, 1993; Kinugawa et al., 1994). Similarly, it has been shown that TNF-α has a direct negative inotropic effect on contractile function in human, rat, hamster and dog myocardium (Finkel et al., 1992; Cain et al., 1998, 1999; Rathi et al., 2002; Thielmann et al., 2002). This appears to be mediated through altered Ca^2+^ handling, manifested by impaired Ca^2+^-induced Ca^2+^ release and reduced myofilament sensitivity to Ca^2+^ (Saini et al., 2005). Finally, it must be noted that efficient early inflammation is required for transitioning to the resolution phase, which could explain the conflicting results in the literature showing pro-inflammatory cytokines as both needed for and deleterious to cardiac repair and regeneration (Prabhu and Frangogiannis, 2016).

A decrease in interstitial fibrosis was also observed in the MA-treated group. Interstitial fibrosis usually leads to increased myocardial stiffness and diastolic dysfunction (Zile et al., 2015). Moreover, excessive collagen deposition might negatively impact systolic function of the left ventricle by hindering cardiomyocyte force transmission and perfusion, impairing excitation-contraction coupling, disrupting the link between ECM and the sarcomeric contractile apparatus, and activating interstitial cells that secrete factors suppressing cardiomyocyte function (Frangogiannis, 2019; Iwanaga et al., 2002). Thus, decreasing interstitial fibrosis could be one of the mechanisms behind the improved systolic function of the left ventricle we observed at 5 weeks post myocardial infarction in the MA-treated group.

Stimulation of angiogenesis is one of the main mechanisms of MSC-mediated tissue regeneration (Gomez-Salazar et al., 2020), and is mainly mediated by secretion of proangiogenic factors, such as fibroblast growth factor (FGF), vascular endothelial growth factor (VEGF), platelet-derived growth factor (PDGF), angiopoietin, and others. Many such factors are upregulated with hypoxia, indicating a possibility of increased expression upon reaching the ischemic site of injury (Kinnaird et al., 2004). Here, we showed that the MA-treated group had increased blood vessel density in the border zone of the infarct. Moreover, by using HUVEC tube formation and wound healing assays, we showed the ability of blood-derived MSCs to support angiogenesis *in vitro*. This observation was not only in support of our *in vivo* results but also provided new evidence that blood-derived MSCs support angiogenesis *in vitro*, a finding that, so far, has mainly been reported for *ex vivo* expanded tissue-resident cells, such as bone marrow- and adipose tissue-derived MSCs (Maacha et al., 2020).

Our data propose several insights into the molecular mechanisms that regulate myocardial infarction-induced MSC mobilisation and its pharmacological enhancement. The CXCR4 antagonist AMD3100 has the unique ability to reverse the gradient of CXCL12 across the bone marrow endothelium, causing an acute increase in plasma levels of CXCL12. This phenomenon was shown to be crucial for HSPC and MSC mobilisation from the bone marrow (Fellous et al., 2020; Redpath et al., 2017; Dar et al., 2011) and, recently, the molecular pharmacology underlying this response has been elucidated (Jørgensen et al., 2021). Our previous work has shown that the pharmacological mobilisation of MSCs is a two-step process, with the first step involving β_3_AR agonism in the bone marrow, leading to increased local production of endocannabinoids and N-acyl ethanolamines, whereas the second step – driven by acute administration of AMD3100 – required the aforementioned reversal of the CXCL12 gradient across the bone marrow endothelium (Fellous et al., 2020). In line with this observation, we showed in this study that myocardial infarction-induced mobilisation of endogenous MSCs at day 5 post injury coincided with a significant increase of plasma CXCL12, suggesting involvement of this chemokine in the physiological mobilisation process. The plasma CXCL12 levels were further elevated after AMD3100 treatment, in both the MA and A groups. Moreover, the MA and A groups also had elevated circulating HSPC levels compared to vehicle, but only the MA group had elevated circulating MSC levels, consistent with our previous work showing that pre-treatment with mirabegron is necessary for MSC mobilisation (Fellous et al., 2020). Finally, we recorded the lowest levels of circulating MSCs in the β3-AR antagonist (SR59230A)-treated group, although no significant inhibition of myocardial infarction-induced MSC mobilisation was present. This observation warrants further research. However, we showed here that both CXCL12 and β3-AR signalling are needed, and neither is sufficient for the pharmacological enhancement of myocardial infarction-induced MSC mobilisation, as mirabegron only or AMD3100 only did not result in significant increase of circulating MSCs.

MSCs were identified by their CFU-F-forming ability, morphology, cell surface marker expression and ability to undergo trilineage differentiation. It is noteworthy that the circulating MSCs exhibit all these characteristics, except for differentiation into adipocytes. This suggests that circulating MSCs are osteochondral progenitors or another distinct subset of MSC progenitors. Future work will be required to more thoroughly investigate the similarities and differences between tissue-resident and circulating MSCs by using RNA-seq or other ‘omics’ approaches.

A limitation of our study is the lack of definitive evidence that mobilised MSCs are, indeed, trafficking to the site of the myocardial injury. Owing to the lack of a specific marker for rat MSCs and the limited understanding of their *in vivo* identity, it is currently impossible to track these cells *in vivo*. Nevertheless, the functional effects we have observed are consistent with the known functions of MSCs.

In conclusion, we have shown that endogenous stem/progenitor cells (MSCs and HSPCs) mobilise into the blood with distinct kinetics in response to myocardial infarction. We demonstrated that this myocardial infarction-induced MSC mobilisation can be enhanced at day 5 post myocardial infarction using a combination of two drugs approved by the FDA, i.e. mirabegron and AMD3100 (MA). The group receiving this treatment had improved cardiac contractility at 5 weeks post myocardial infarction and enhanced blood vessel density in the border zone of the infarct, decreased interstitial fibrosis, and reduced levels of the plasma pro-inflammatory cytokines (IL-6 and TNF-α). Based on this report, we suggest a novel, non-invasive, cost-efficient and easily translatable regenerative pharmacological approach for improving cardiac function after myocardial infarction.

## MATERIALS AND METHODS

### Animal care and procedures

Male Lewis rats (*rattus norvegicus*) purchased from Charles River Laboratories were used for all experiments. Rats weighed between 250 and 300 g. To create the rat myocardial infarction model permanent ligation of the left anterior descending artery (LAD) surgery was performed as described before (Prabhu and Frangogiannis, 2016). For this, animals were anaesthetised using 5% isoflurane and anaesthesia was maintained using 2-3% isoflurane. For local anaesthesia around the site of the initial incision, four injections of 10 µl bupivacaine hydrochloride and epinephrine (Marcaine 0.5%) were administered. Following surgery, carprofen (5 mg/kg/day) and buprenorphine (0.05 mg/kg/day) were administered subcutaneously for pain control during the first 2 days after surgery. Animal housing and treatment was done in accordance with the Animals (Scientific Procedures) Act 1986 Amendment Regulations 2012, and European Union directive 2010/63/EU.

To study post myocardial infarction progenitor mobilisation, we evaluated four timepoints, i.e. day 1, 3, 5 and 10, after myocardial infarction (*n*=4 for each timepoint) (Fig. S1). At each timepoint, a terminal procedure to collect bone marrow and peripheral blood was performed. The samples were used for quantification of stem/progenitor cells and total nucleated cell frequency, and CXCL12 levels. For the drug selection study, all rats underwent myocardial infarction surgery and were assigned to one of five treatment groups, i.e. vehicle (*n*=7), mirabegron only (M) (*n*=8), AMD3100 only (A) (*n*=6), mirabegron+AMD3100 (MA) (*n*=10) or SR59230A (SR) (*n*=6). Rats were then given the corresponding drugs and, on day 5, bone marrow and peripheral blood were collected for analysis (Fig. S2). The plasma from these animals was used for the day 5 timepoint for cytokine and chemokine assessment. The final animal study was the long-term evaluation of the effect of mirabegron+AMD3100 (MA) (*n*=7) on cardiac function compared to vehicle (*n*=6) (Fig. S3), which assessed cardiac function by using blinded cardiac MRI analysis at two timepoints post myocardial infarction, i.e. day 5 (acute) and 5 weeks (chronic). Animals were sacrificed after performing the chronic timepoint MRI, and blood and bone marrow were collected for analysis.

### Cell culture, quantification and characterisation of stem/progenitor cells

To isolate the mononuclear cells from the blood, the Ficoll-Paque density gradient centrifugation method was used as per manufacturer's instructions. Bone marrow cells were isolated from femurs and the red blood cells (RBCs) were lysed using the RBC lysing solution (eBioscience). For the CFU-F assay, blood mononuclear or bone marrow cells were plated at 0.32×10^6^/cm^2^ in Dulbecco's modified Eagle’s medium (DMEM), supplemented with 20% foetal bovine serum (FBS), and cultured for 7 days prior to the first change of medium. Thereafter, medium was changed 2-3 times a week. Fibroblast-like colonies were manually counted under a light microscope on day 14, after seeding in a blinded fashion. Colonies were expanded in culture for flow cytometry and differentiation analysis (Table S1, Figs S4, S5 and S7). For quantification of HSPCs, MethoCult™ GF R3774 medium was used as per the manufacturer's instructions (StemCell). CFUs were manually counted on day 10 post seeding in a blinded fashion.

### Quantification of CXCL12, IL-6, TNF-α and IL-10

Protein concentrations of chemokine CXCL12 and cytokines IL-6, TNF-α and IL-10 were analysed using the sandwich ELISA method (see Supplementary Materials and Methods).

### Quantification of blood vessel density and interstitial fibrosis

Details about preparation of heart tissue for histology are included in the Supplementary Materials and Methods, together with the methods for the quantification of blood vessel density using isolectin-B4 and interstitial fibrosis using Masson's trichrome stained heart sections (see Supplementary Materials and Methods).

### *In vitro* angiogenesis assays

HUVECs were purchased from Lonza and used between passages 1 and 6. For tube formation assays, we used the Ibidi µ-Slide Angiogenesis slides as per the manufacturer's instructions. Example images of tube formation assays are shown in Fig. S11. Wound healing assays were performed as described previously (Ong et al., 2018), and example images are shown in Fig. S11B. Detailed protocols can be found in the Supplementary Materials and Methods. Images were taken on a widefield microscope (AxioObserver Z1) and analysed using ImageJ. For tube formation assays, we used an Angiogenesis Analyser macro developed for ImageJ (Higuchi et al., 2007).

### Cardiac MRI

Cine MRI was used to assess global heart function and LGE for quantifying myocardial scar size on day 5 and week 5 post myocardial infarction (see Supplementary Materials and Methods). Data analysis was performed by two independent users in a blinded fashion, using а semi-automated left ventricle segmentation software in the freely available program Segment (Ponikowski et al., 2016; Odeh, 1993).

### Statistical analysis

For statistical analysis (Student's *t*-tests, one-way ANOVA, two-way ANOVA, normality tests, etc.), we used GraphPad 9.1 software. For all comparisons, *P*<0.05 was considered significant. Data are presented as the mean±standard error of the mean (s.e.m.).

## Supplementary Material

10.1242/dmm.049630_sup1 Supplementary information
