# Supplementary material for: Drug-based mobilisation of mesenchymal stem/stromal cells improves cardiac function post myocardial infarction
Source: Dis Model Mech. 2022 Nov 4;16(5):dmm049630. doi: 10.1242/dmm.049630 (PMC10655717; doi:10.1242/dmm.049630)
Supplement: Supplementary information [file dmm-16-049630-s1.pdf]

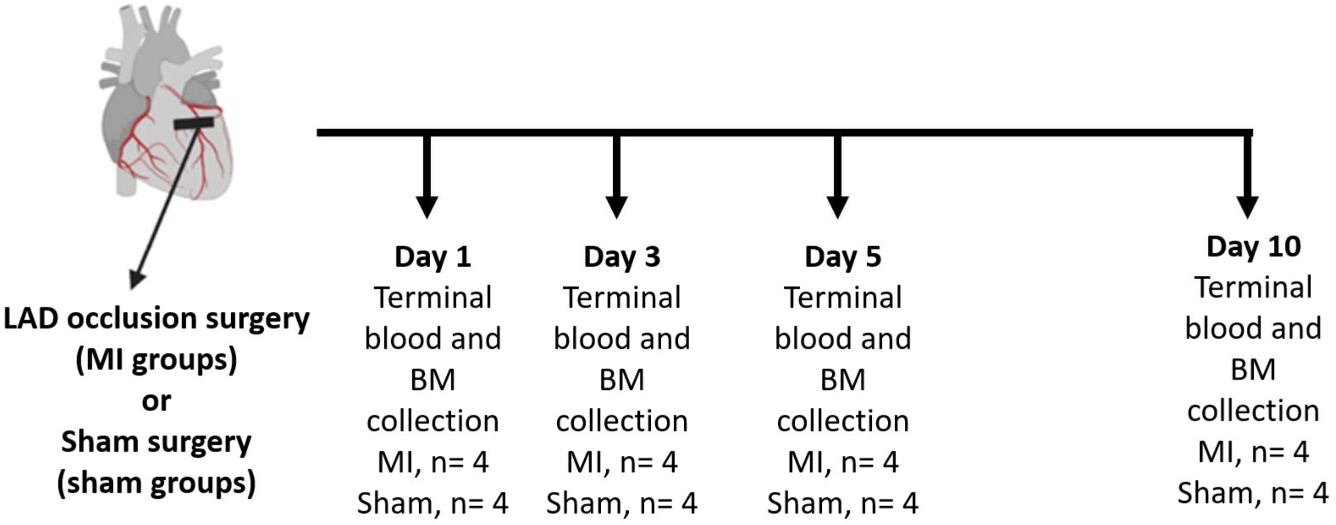

Fig. S1. Schematic representation of the experimental design for the post MI stem cell mobilisation study. A total of 32 animals were used for these experiments.

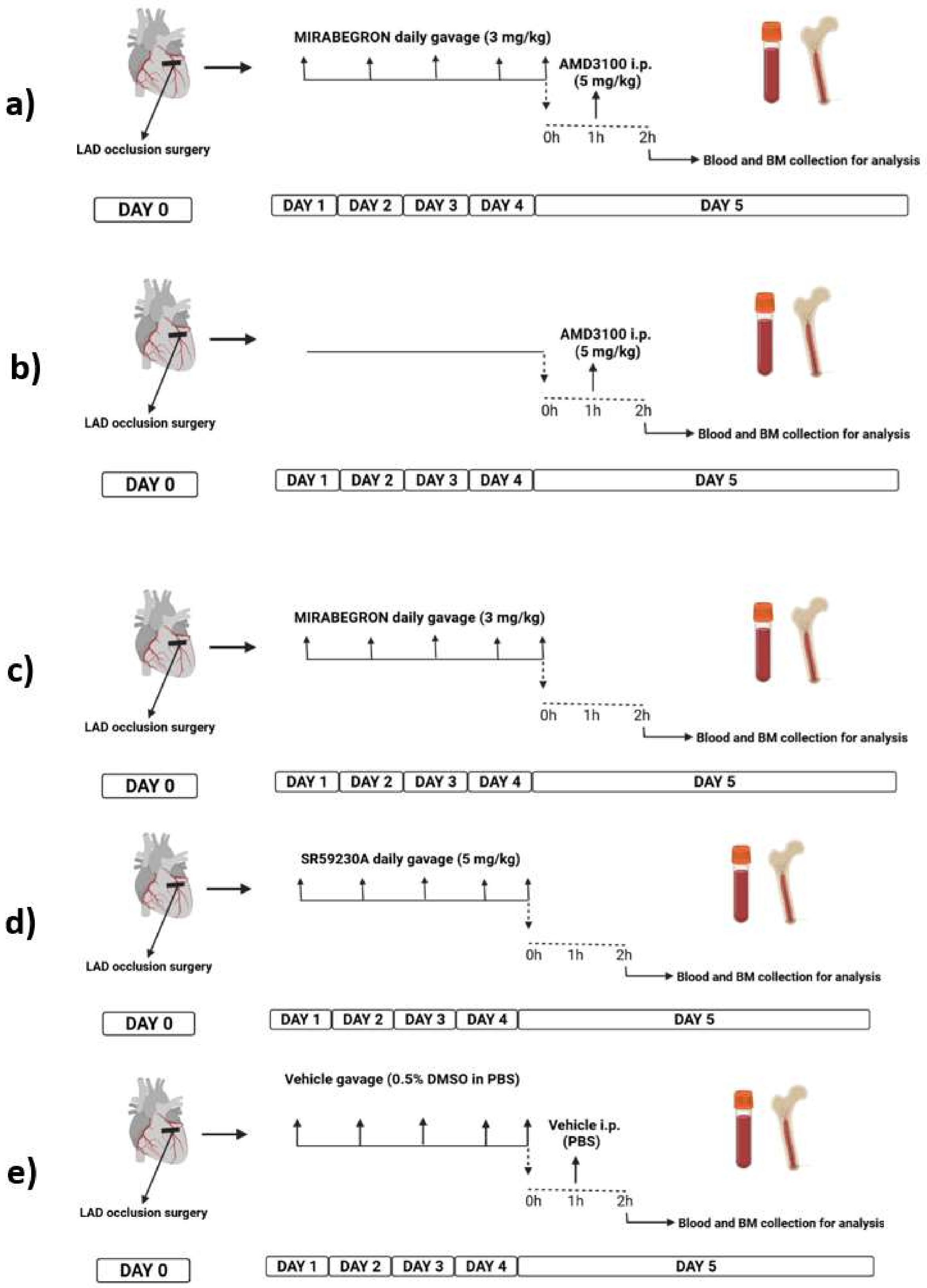

Fig. S2. Schematic representation of the drug administration regimens following MI surgery for the study of pharmacological modulation of stem cell mobilisation. A) Mirabegron+AMD3100 (MA) drug administration. B) AMD3100 only (A) drug administration. C) Mirabegron only (M) drug administration. D) SR59230A (SR) drug administration. E) Vehicle (V) group drug administration. Created with Biorender.com

Disease Models & Mechanisms • Supplementary information

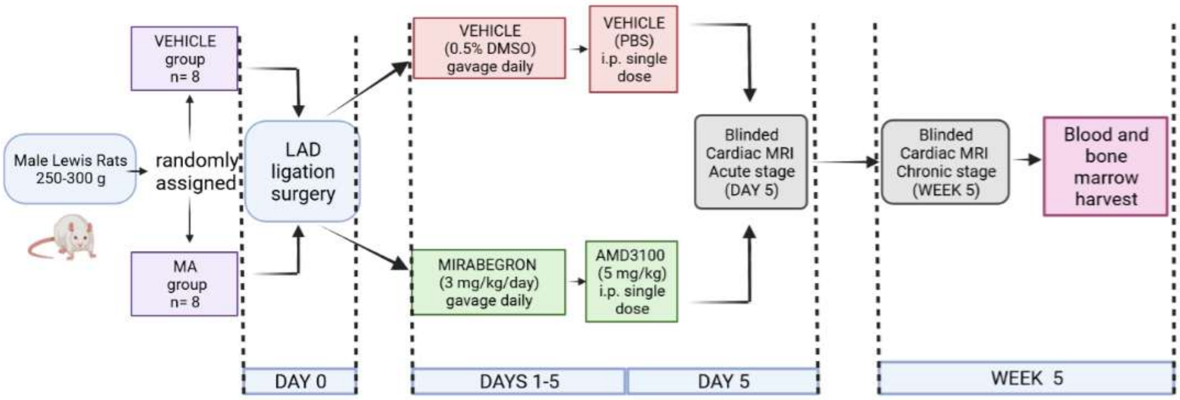

Fig. S3. Study design for assessment of the effect of mirabegron+AMD3100 treatment on cardiac function after MI. One rat from each group died within 24h of surgery, reducing the n number from 8 to 7 for each group. One rat from the vehicle group was excluded due to lack of significant infarct (<10%), reducing the vehicle group to n= 6. (Created with BioRender.com)

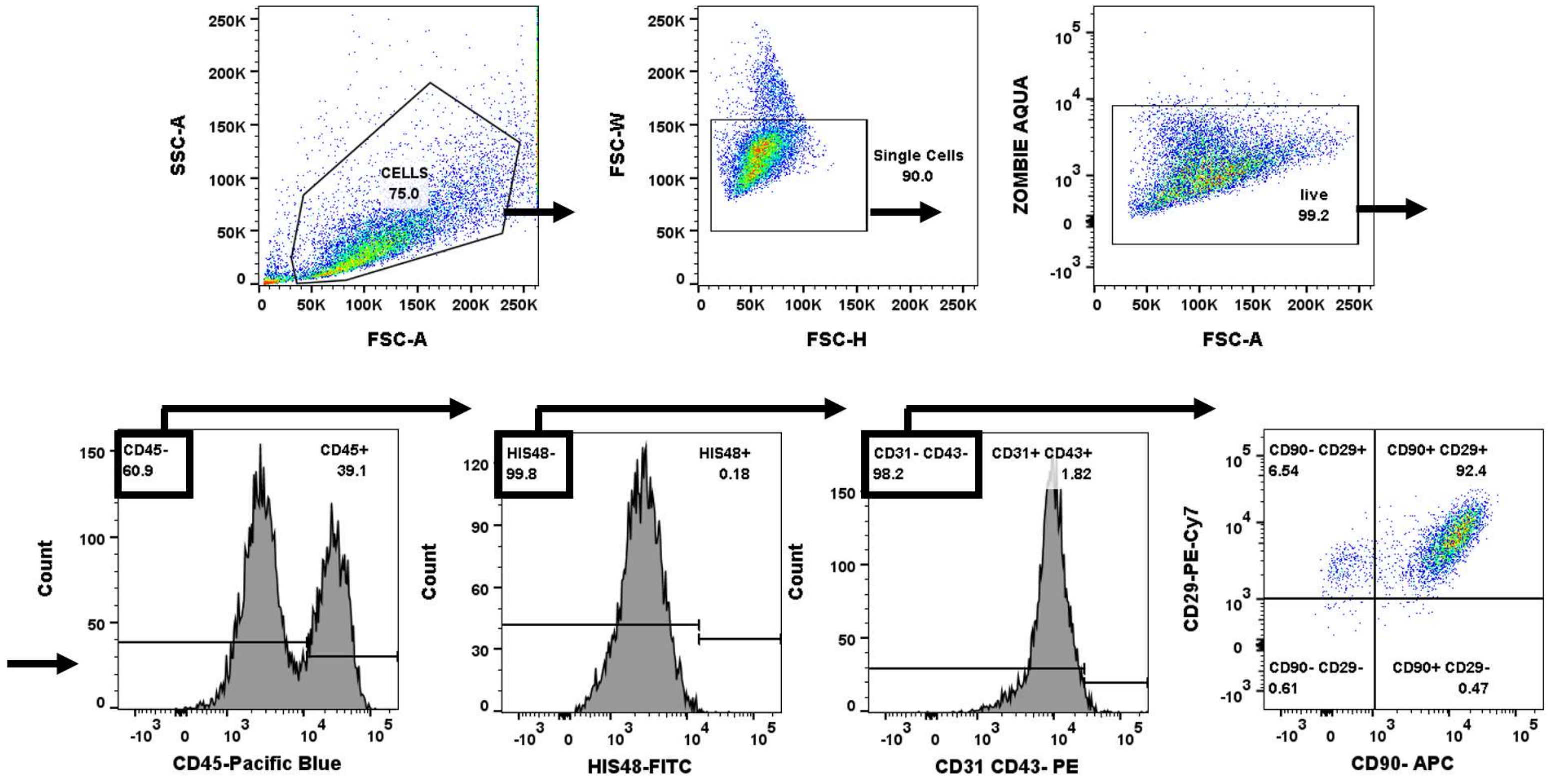

Fig. S4. Gating strategy for the flow cytometry analysis of BM and blood derived culture expanded CFU-Fs. From left to right, top to bottom: first we gated into whole cells to exclude debris (FSC-A, SSC-A), then the single cells were gated into (FSC-H, FSC-W), followed by the live cells (Zombie Aqua™ negative). These were then gated into the CD45-negative fraction, to exclude haematopoietic cells, followed by exclusion of the HIS48+ and CD31+ CD43+ to remove any granulocytes, endothelial cells, and monocytes. Finally, we looked at the CD90 and CD29 expression to determine the rat MSC cell surface marker expression.

Disease Models & Mechanisms • Supplementary information

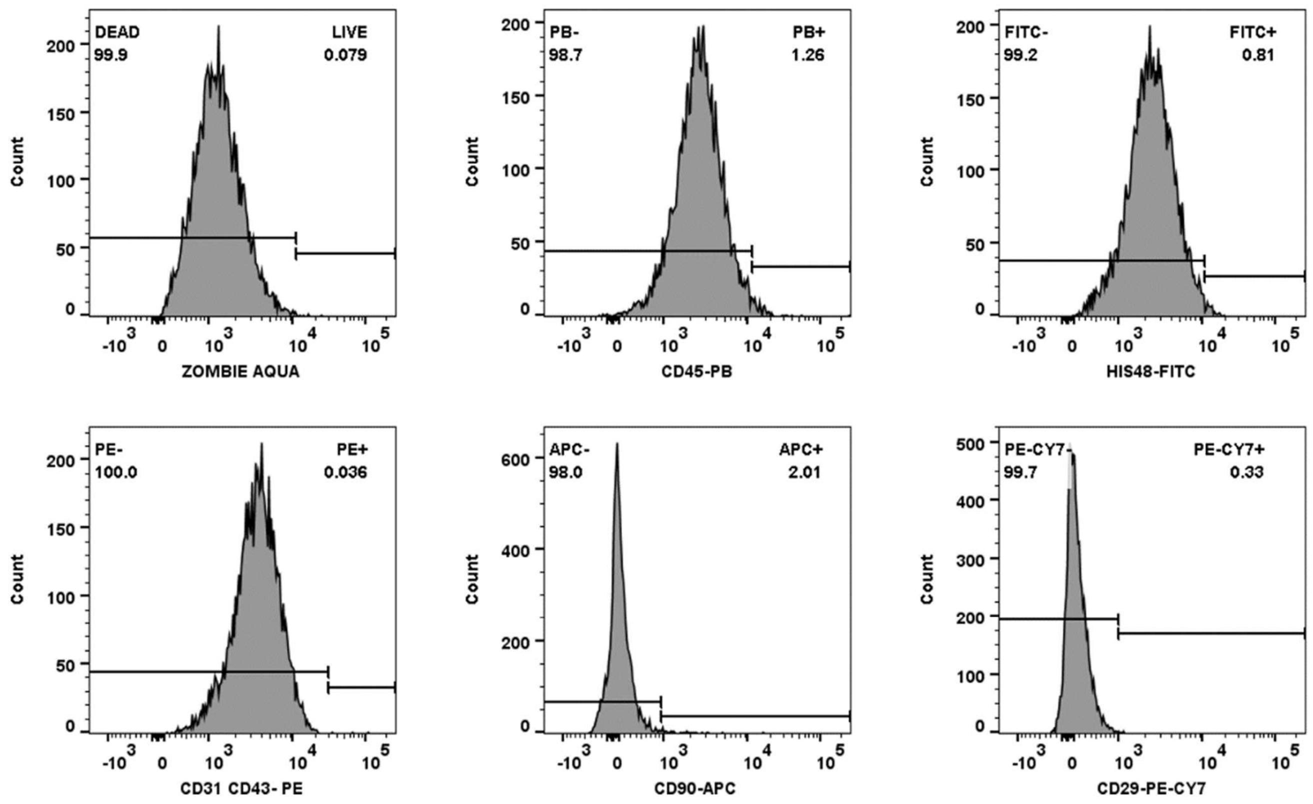

Fig. S5. Fluorescence minus one (FMO) controls for the flow cytometry analysis. Here we show the gates we used in our analysis, based on the FMO controls, which included all but one marker to ensure that the appropriate negative population is being gated out. From left to right, top to bottom we show the gates for Zombie Aqua (live/dead), Pacific Blue (CD45), FITC (HIS48), PE (CD31, CD43), APC (CD90), PE-Cy7 (CD29).

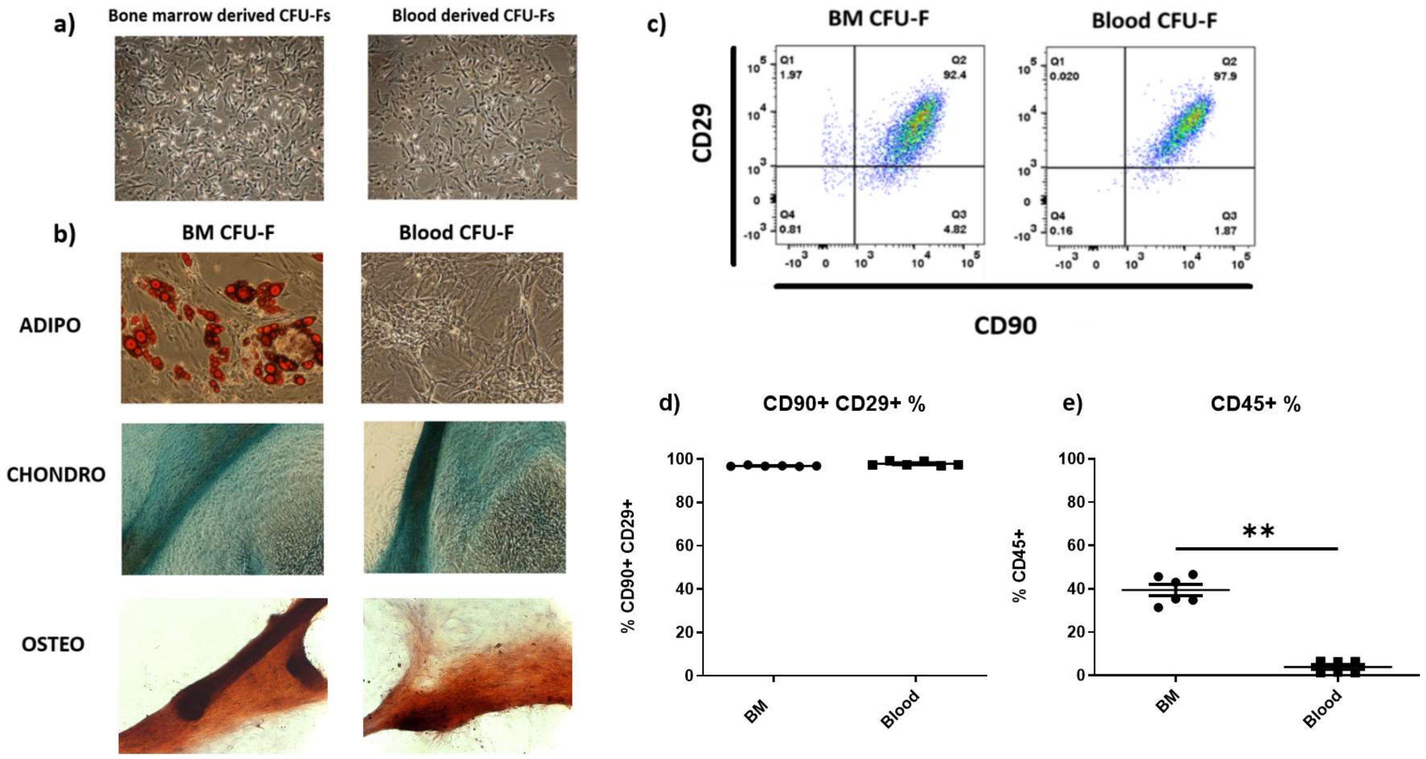

Fig. S6. MSCs characterisation. A) Blood and BM derived CFU-Fs were fibroblast like and plastic adherent in culture. Brightfield image, 10X. B) Blood and BM derived CFU-Fs were culture expanded (P2-P8) and induced to differentiate into adipo-, chondro- and osteocytes. Culture expanded CFU-Fs from the BM (vehicle and SR groups), but not from the blood and BM (MA, M, A groups), differentiated into adipocytes (fat droplets stained red by Oil Red-O), both BM and blood derived CFU-Fs differentiated into chondrocytes (glycosaminoglycans stained blue by Alcian Blue) and osteocytes (calcium deposits stained in brick red by Alizarin Red S). C) Blood and BM culture expanded (P2-P8) CFU-Fs were double positive for CD90 and CD29 (gated on live, single, CD45-, CD43-, CD31- , His48- cells). D) Percentages of double positive CD90+ CD29+ BM and blood derived CFU-Fs (gated on live, single, CD45-, CD43-, CD31- , His48) (Unpaired t test, p= 0.0562, n=6) E) Percentage of CD45+ cells in BM and blood derived CFU-Fs (gated on live, single cells) (Mann-Whitney test, p= 0.0022, n=6)

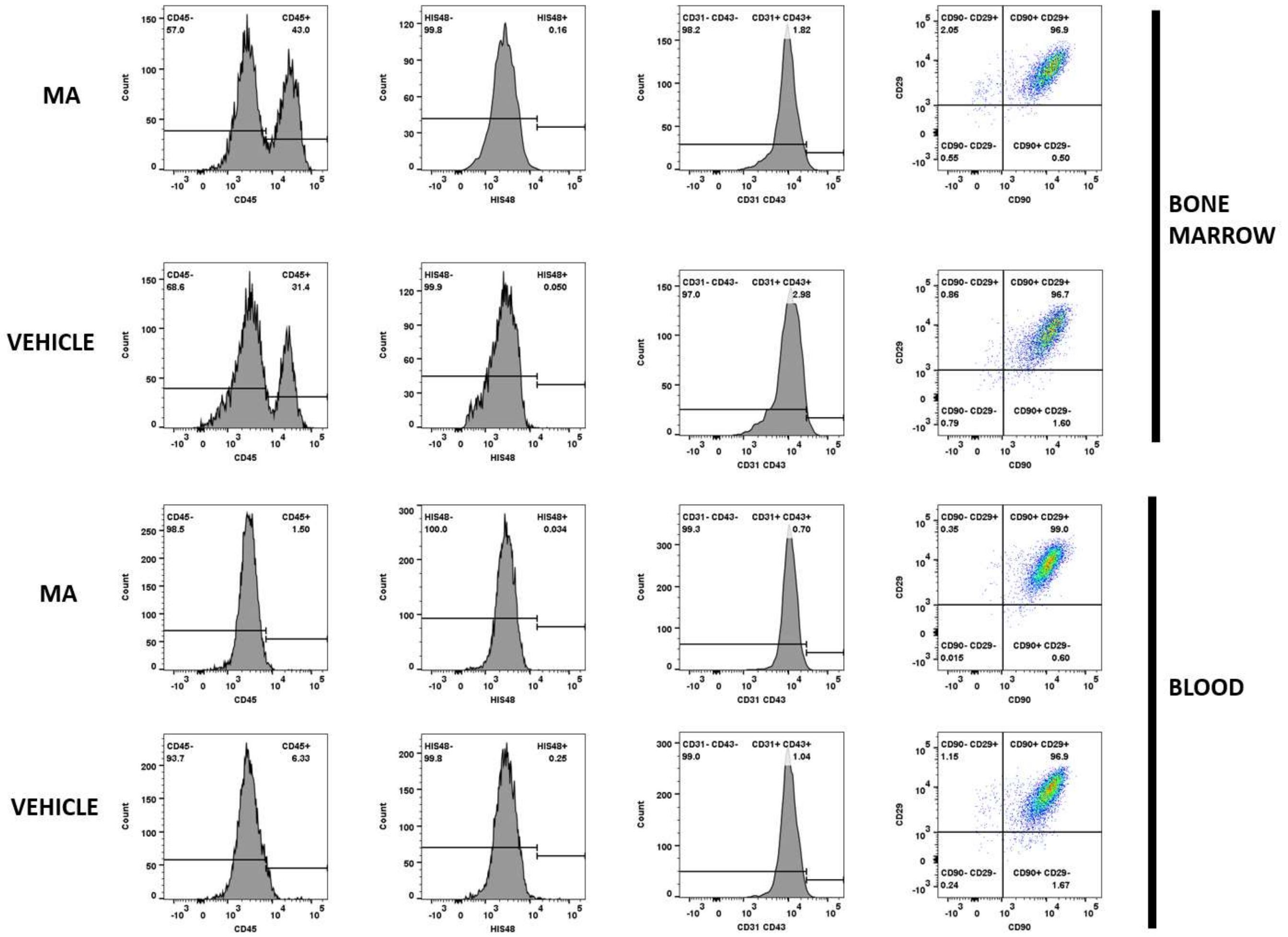

Fig. S7. Representative histograms and scatter plots of the flow cytometry data. Here we have shown representations of the flow cytometry data for the BM and blood derived culture expanded CFU-Fs (MSCs) from both groups: vehicle and MA. The gating is shown in the Supplementary Methods.

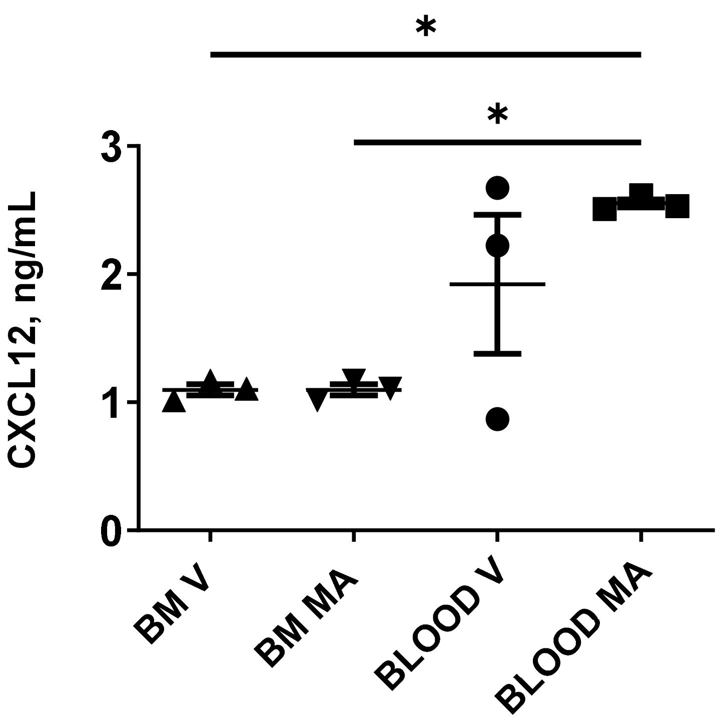

Fig. S8. CXCL12 levels in conditioned medium (CM) from blood and BM derived MSCs. This graph shows the CXCL12 concentration (ng/mL) in the CMs from culture expanded blood and BM MSCs from the vehicle and MA groups. The data were obtained using the sandwich ELISA method. (Ordinary One-Way ANOVA, Tukey post-test, * $p < 0.05$, $n = 3$)

Disease Models & Mechanisms • Supplementary information

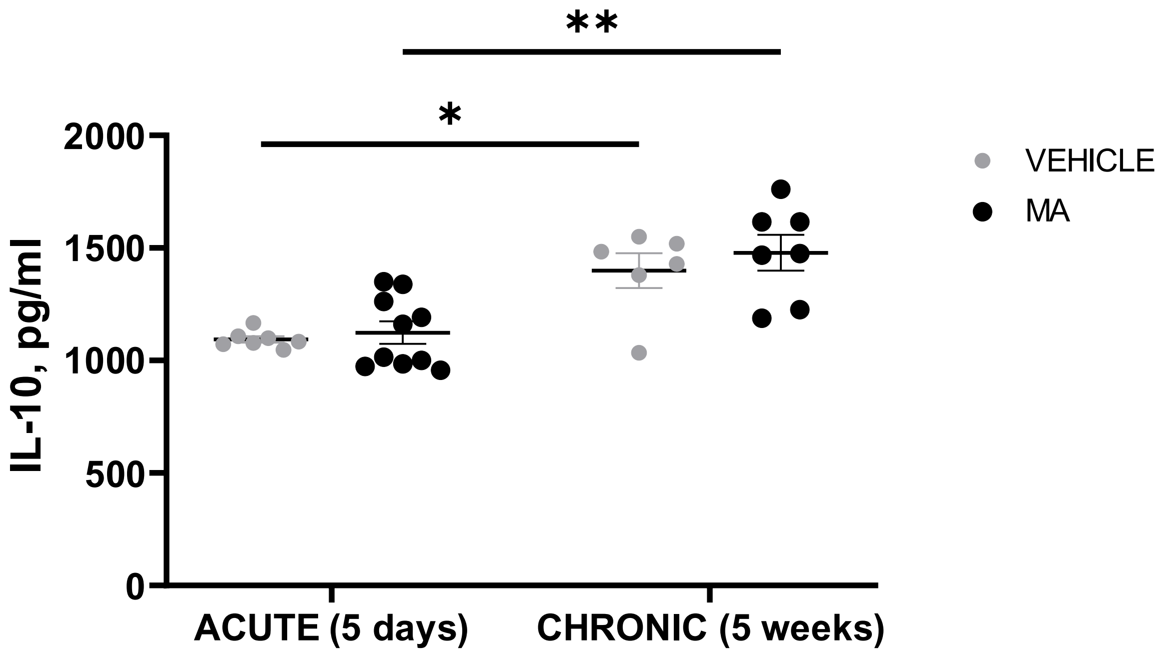

Fig. S9. IL-10 levels in the plasma of the vehicle and MA treated groups at 5 weeks post MI. The IL-10 concentration is given as pg/mL, Two Way ANOVA, Sidak's multiple comparisons test, * $p < 0.05$, ** $p < 0.01$, vehicle n= 6, MA n= 7

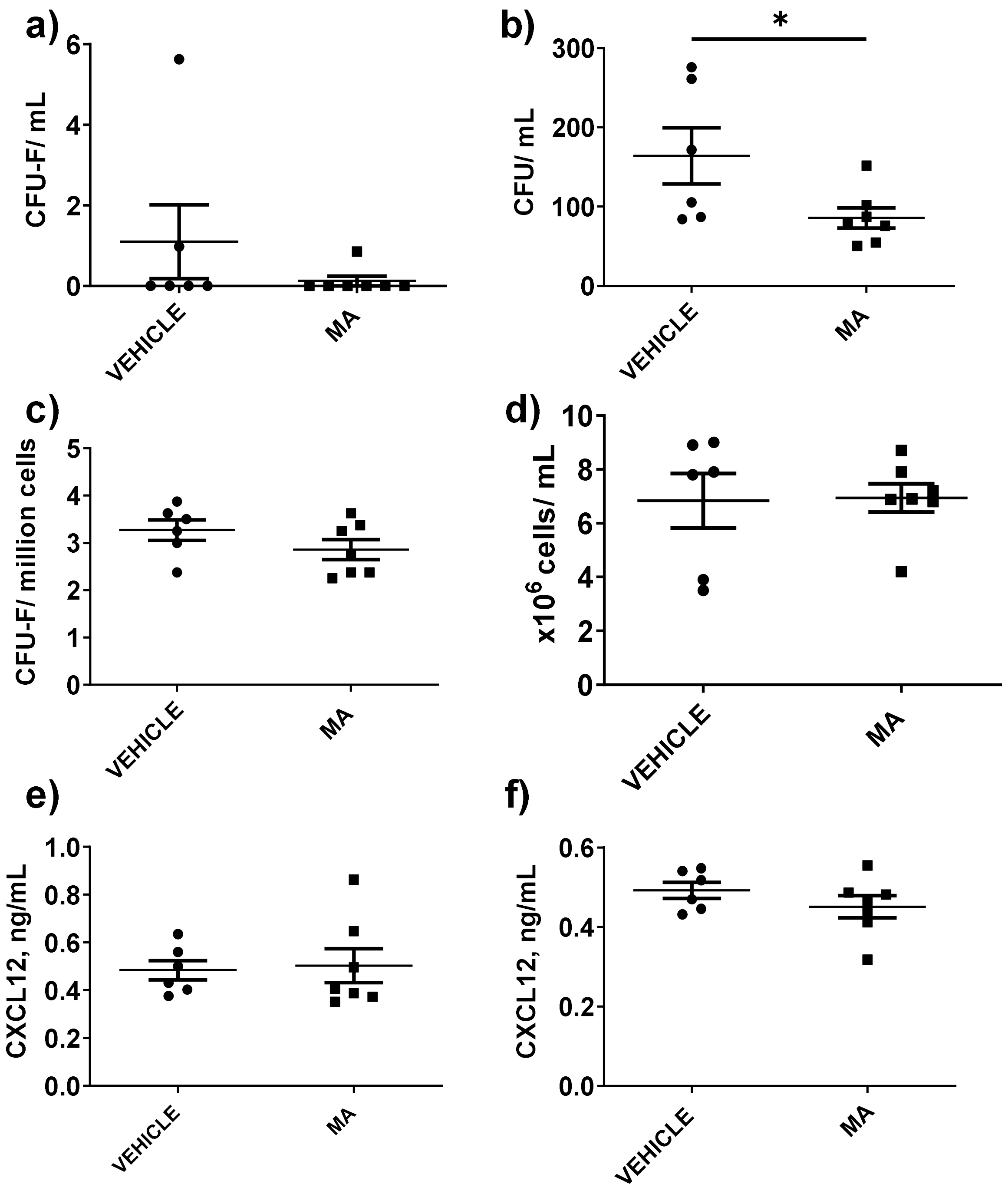

Fig. S10. Stem/progenitor mobilisation dynamics at 5 weeks post MI. This graph summarises the stem/progenitor mobilisation dynamics in the vehicle (n= 6) and MA group (n= 7). A) Circulating MSPCs are represented as CFU-F/mL (Mann-Whitney test, p= 0.3147). B) Circulating HSPCs are shown as CFU/mL (Unpaired t-test, p= 0.0494). C) BM MSPC numbers are shown as CFU-F/ million cells (Unpaired t-test, p=0.2003). D) Circulating total nucleated cells are shown as million cells/mL (Unpaired t-test, p=0.9229). Plasma (E) and BM (F) CXCL12 are shown as ng/mL, (Mann-Whitney test, p= 0.7308 and p= 0.4452, respectively)

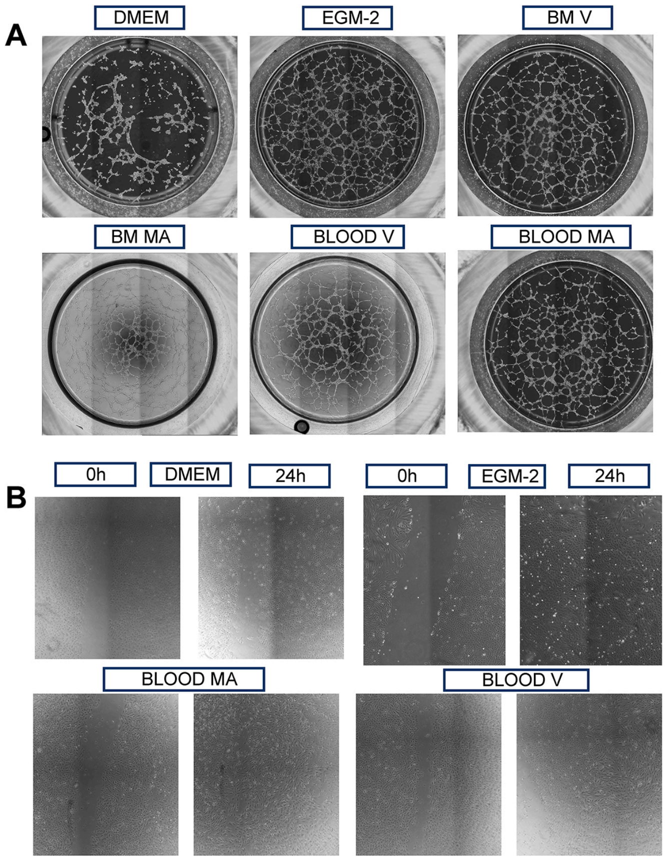

Fig. S11. Example images of tube formation assay and wound healing assay. (A) Example images from the tube formation assay are given for each group: HUVECs treated with DMEM (negative control), EGM-2 (positive control), conditioned medium from blood-derived rat MSCs (rMSCs) from the MA group (BLOOD MA), conditioned medium from blood-derived rMSCs from the Vehicle group (BLOOD V), conditioned medium from bone marrow-derived rMSCs from the MA group (BM MA), conditioned medium from BM-derived rMSCs from the Vehicle group (BM V). (B) Example images from the wound healing assay are given for HUVECs treated with DMEM (negative control), EGM-2 (positive control), conditioned medium from blood-derived rMSCs from the MA group (BLOOD MA) and conditioned medium from blood-derived rMSCs from the Vehicle group (BLOOD V). The time after producing the scratch (‘wound’) is indicated as 0 h (immediately after) and 24 h (24 h after).

Disease Models & Mechanisms • Supplementary information

Table S1. List of fluorophore-conjugated antibodies used in our analysis of culture expanded bone marrow and blood derived CFU-F.

Fluorophore	Rat Antigen	Clone	Manufacturer
Pacific Blue^{TM}	CD45	OX-1	Biolegend
PE	CD43	W313	Biolegend
PE	CD31	TLD-3A12	BD Biosciences
APC	CD90	OX-7	Biolegend
PE-Cy7	CD29	HMb1-1	Biolegend
FITC	Granulocyte marker	HIS48	Invitrogen
Zombie Aqua	Dead cells	-	Biolegend
n/a	CD32, Fc block	D34-385	BD Biosciences

Supplementary Materials and Methods

Animal studies: experimental design and drug administration

For the study investigating the natural stem cell mobilisation post MI, Lewis rats in the MI group underwent left anterior descending artery (LAD) ligation surgery, and the animals in the sham group underwent a sham surgery, which did not include tying of the suture around the vessel. For each timepoint we assessed 4 animals in each group (MI and sham), and we had a total of four timepoints: day 1, 3, 5, and 10 (Fig. S1). The rats were culled at each of these endpoints for collection of peripheral blood (PB) by cardiac puncture and hind legs for bone marrow (BM) extraction.

For the drug selection study, all rats underwent MI inducing surgery and were randomly assigned to five groups: vehicle (n= 8), mirabegron only (M, n= 8), AMD3100 only (A, n= 8), mirabegron+AMD3100 (MA, n= 12), and SR59230A (SR, n= 8). Only two surgeries a day could be performed, meaning that the experiments were spaced out in time. Thus, we decided to include additional animals in the MA group to maximise the probability of them being more evenly distributed throughout the study and to also account for potential loss of animals, as this was the main group of interest. The surgeon was blinded to the group assignment. To the MA group, mirabegron (YM178, ApexBio) was given at 3 mg/kg/day using the gavage method for five days post MI starting at 24h after surgery, and after 5 days AMD3100 (AMD3100 octahydrochloride hydrate, Sigma) was administered, one hour after the last dose of mirabegron, at 5 mg/kg using the intraperitoneal injection method (Fig. S2a). The A group received only the single dose of AMD3100 (5 mg/kg) (Fig. S2b), the M group received only the daily gavage of mirabegron (3 mg/kg) (Fig. S2c), and the SR group received daily gavage of the β_3AR antagonist SR59230A (SR 59230A hydrochloride, Tocris) (5 mg/kg) for the first five days after MI (Fig. S2d). The vehicle group received 0.5% dimethylsulfoxide (DMSO) for 5 days (gavage) and a single dose (i.p.) of phosphate buffered saline (PBS) on the last day (Fig. S2e). The BM and PB samples were collected 1h after the final drug injection on day 5 for stem/progenitor cell enumeration and cytokine and chemokine level analysis. As the scar sizes were estimated using Masson's trichrome, all animals that had a scar size of less than 10% were excluded: vehicle (n= 1), AMD3100 only (n= 2), mirabegron+AMD3100 (MA, n= 2) and SR59230A (n= 2), which lead to the final n numbers of: vehicle (n= 7), mirabegron only (M, n= 8), AMD3100 only (A, n= 6), mirabegron+AMD3100 (MA, n= 10) and SR59230A (SR, n= 6).

Disease Models & Mechanisms • Supplementary information

The final animal study looked at the effect of mirabegron+ AMD3100 (MA) on cardiac function at 5 weeks after MI surgery. The experimental design is summarised in Figure S3 below. The drug administration regimens for the MA and the vehicle groups were the same as in the drug selection study described above (Fig. 2a, e). Briefly, MI inducing surgery was performed on Lewis rats, which were randomly assigned to two groups: vehicle and MA. The animal surgeon was blinded to group assignment at the time of surgery. Each group then received the corresponding treatment and at day 5 after the last drug administration they underwent the first cardiac MRI (acute stage), which was then followed with the second cardiac MRI 5 weeks later (chronic stage). The animals were culled at the 5-week timepoint following the second MRI scan and the PB, BM and hearts were collected for analysis.

Differentiation assays

Differentiation assays were performed in 24-well plates with cells seeded at $2x10^5$ per well for the adipogenesis and osteogenesis and $4x10^5$ for the chondrogenesis and expanded until confluent in DMEM+GlutaMax™ supplemented with 20% FBS (D20) before changing to the defined differentiation induction media. Media changes were performed every 3-4 days. The adipogenic differentiation medium was α-MEM+ GlutaMax™, supplemented with 10% FBS, 1 uM dexamethasone, 0.2 mM indomethacin, 10 µg/mL insulin, 0.5 mM 3-isobutyl-1-methylxanthine (IBMX), and 3.5 g/L glucose. Fat droplets were stained in red by using Oil-red-O as described previously [1]. The osteogenic differentiation medium was α-MEM+ GlutaMax™, supplemented with 10% FBS, 0.01 µM dexamethasone, 10 mM beta-glycerophosphate, 0.2 mM ascorbic acid-2- phosphate, 3.5 g/L glucose. Calcium deposits were stained in brick red by Alizarin Red S as described previously [1]. The chondrogenic differentiation medium was DMEM+GlutaMax™, supplemented with 10% FBS, 0.1 µM dexamethasone, 50 µg/mL ascorbic acid-2- phosphate, 40 µg/mL L-proline, 1x ITS+3 supplement, 10 ng/ml TGF-beta 3, 1% sodium pyruvate, 4.5 g/L glucose. Glycosaminoglycans were stained in blue by using Alcian Blue as described previously [1].

Flow cytometry analysis

Flow cytometry analysis was done on cultured plastic adherent BM and blood CFU-F derived cells to assess their expression of the cell surface markers CD45, HIS48, CD31, CD43, CD90 and CD29, which were selected based on previous work that shows CD90+ CD29+ CD45- CD31- HIS48- CD43- to be a rat MSC marker panel [2-5]. The list of antibodies we used is included in Table S1 below. All steps were done on ice and flow buffer (2% FBS in PBS) was used for all staining except the live/dead stain Zombie Aqua™, which was done in PBS only. Cells were pelleted in a 96-well plate for the staining procedure. Unspecific binding was prevented using Fc block (1:200) prior to staining. After this, samples were stained with Zombie Aqua™ (1:200) for 10 min to separate dead cells from the analysis. Finally, the fluorophore-conjugated antibodies were added to the cells and incubated in the dark for 30 min. The cells were washed and resuspended for analysis on the LSR Fortessa II. We used the following controls: compensation (single stains) and 'fluorescence minus one' (FMO) controls. Compensation was done manually using the single stained cells. The gating strategy is summarised in Fig. S4 and the FMO controls are shown in Fig. S5. We used the FMO controls to set the negative gates for the analysis.

Sandwich ELISAs for CXCL12, TNF-alpha, IL-6, and IL-10

The protein concentrations of the chemokine CXCL12 and the cytokines IL-6, TNF-alpha and IL-10 were analysed using the sandwich ELISA method. All antibodies were acquired from Peprotech. Briefly, the plates were coated with the capture antibodies overnight and were washed using washing solution (WS, 0.5% Tween in PBS) prior to blocking with blocking solution (BS,10% FBS in PBS) for an hour. The plates were then washed with WS, and the plasma or BM supernatant samples were added and incubated overnight. Following another wash step, the primary antibodies conjugated

Disease Models & Mechanisms • Supplementary information

to biotin were added for an hour. After this, plates were washed and the streptavidin-HRP was added for 30 min. Plates were washed again and the TMB substrate was added until full colour development, after which the reaction was stopped with sulphuric acid (1 mol/L). Results were read in a plate reader at 450 nm.

Histology

Following the sacrifice of the animal, the heart was excised, washed in heparinised PBS, and fixed in 4% Histofix overnight. Following fixation, the hearts were stored in 70% ethanol. The hearts were embedded in wax, 4 μm sections were obtained and Masson's trichrome staining was performed. Immunofluorescent staining was performed on heart sections according to the following protocol: sections were dewaxed in neoclear for 20 min, then rehydrated in increasing percentages of ethanol - 100% (10 min), 95% (5 min), 70% (5 min), 30% (5 min), distilled water (5 min). Following this, antigen retrieval was done by boiling the slides in citrate buffer (pH= 6) for 20 min. The slides were then permeabilised using 0.1% Triton/PBS for 10 min and following this were rinsed in tap water for 5 min. The sections were coated with blocking solution for 1h prior to addition of the primary antibodies. These were diluted in the same blocking solution (10% normal goat serum in 0.1% BSA in PBS). Antibodies were allowed to incubate overnight at 4°C. For the isolectin-B4 staining we used isolectin-B4 conjugated to biotin. Three 10-minute washes in PBS were performed prior to adding the secondary antibodies (streptavidin- Alexa Fluor™ 555) were added and incubated overnight in the same way. Finally, the sections were stained with DAPI and mounted using ProLong™ Gold Antifade Reagent. Images were acquired using AxioObserver Z1 and the data was analysed using ImageJ, with a customised macro for blood vessel density quantification. For the scar size and interstitial fibrosis quantification, we used the Masson's trichrome images of the 5 weeks post MI heart

sections, and a macro was written for both types of analysis. For the scar size quantification, the whole section was analysed, and for the IF, the percentage collagen was calculated against the whole tissue measured: LV and border zone regions were selected manually. For all histology, we performed blinded analysis using a total of 6 levels of the heart, spaced 250 µm.

In vitro angiogenesis assays

Human umbilical vein endothelial cells (HUVECs) were purchased from Lonza and were used between passage 1-6. For the tube formation assays, we used the Ibidi µ-Slide Angiogenesis slides as per manufacturer's instructions. The wound healing assay was performed as described previously [6]. Images were taken on a widefield microscope (AxioObserver Z1) and analysed using ImageJ. For the tube formation assay we used an Angiogenesis Analyzer macro developed for ImageJ [7].

To obtain the conditioned media (CMs), the blood and BM derived culture expanded CFU-Fs (MSCs) were allowed to become confluent in a T25 flask and were then washed with serum-free DMEM and fed serum free DMEM for 24 hours. The CMs were then aspirated and filtered through a 0.2-µm filter for use in the tube formation and wound healing assays.

Cardiac MRI

Rats were anesthetised using 5% isoflurane and maintained under inhalational anaesthesia via a nose cone (2.5% isoflurane). Vital signs and ECG were continuously monitored. All MRI scans were performed on a pre-clinical 9.4 T scanner (94/20 USR Bruker BioSpec; Bruker Biospin, Ettlingen, Germany) housed at the Biological Imaging Centre (BIC), Imperial College London equipped with a volume transmit quadrature coil combined with an actively decoupled rat heart array receiver. Data were acquired

Disease Models & Mechanisms • Supplementary information

with Paravision 6.01 (Bruker, BioSpin). For localisation of the heart, low-resolution ECG and respiratory triggered gradient echo scout scans were acquired in axial, sagittal and coronal orientations followed by pseudo two- and four-chamber gradient echo scans. Left ventricular EF, mass, ESV and EDV were quantified from a stack of ECG and respiratory-gated CINE gradient echo images in the short-axis plane (11-12 slices). The acquisition parameters for CINE measurements were: repetition time (TR)=RR interval/number of frames (~9 ms for 20 frames), TReffective=RR interval, echo time (TE)=2.2 ms, flip angle=18°, slice thickness=1.5 mm (continuous slices), acquisition matrix=190×190, field of view = (38.5×38.5) mm2, leading to a spatial in-plane resolution of (202×202) µm2, scan time: 18-20 min.

Multi slice late gadolinium enhancement (LGE) data were acquired using an inversion recovery gradient echo sequence with a single TI point and flip angle of 90°. The TI was selected to effectively null the healthy myocardium and to provide the best contrast enhancement of the area of infarction. Infarction size (acute stage) and fibrosis (chronic stage) was quantified by tracking the LGE signal for each LV axial slice using the in-built function in the open-source software Segment (version 2.2, Medviso AB, Lund, Sweden). The following acquisition parameters were used: TE = 2.4 ms, TRs = 3.85 ms, FOV = (38 ×38) mm2, matrix size = 180 × 180, spatial resolution (211×211) µm2, 1.5 mm slice thickness, 9-10 slices, scan time: 3 min /slice. All MRI data underwent blinded analysis.

Disease Models & Mechanisms • Supplementary information

Supplementary references

1. Karaoz, E., Aksoy, A., Ayhan, S., Sariboyaci, A.E., Kaymaz, F., Kasap, M., 2009. Characterization of mesenchymal stem cells from rat bone marrow: ultrastructural properties, differentiation potential and immunophenotypic markers. Histochem Cell Biol 132, 533–546. https://doi.org/10.1007/s00418-009-0629-6
2. Miyahara, Y., Nagaya, N., Kataoka, M., Yanagawa, B., Tanaka, K., Hao, H., Ishino, K., Ishida, H., Shimizu, T., Kangawa, K., Sano, S., Okano, T., Kitamura, S., Mori, H., 2006. Monolayered mesenchymal stem cells repair scarred myocardium after myocardial infarction. Nat Med 12, 459–465. https://doi.org/10.1038/nm1391
3. Lotfy, A., Salama, M., Zahran, F., Jones, E., Badawy, A., Sobh, M., 2014. Characterization of mesenchymal stem cells derived from rat bone marrow and adipose tissue: a comparative study. Int J Stem Cells 7, 135–142. https://doi.org/10.15283/ijsc.2014.7.2.135
4. Harting, M., Jimenez, F., Pati, S., Baumgartner, J., Cox, C., 2008. Immunophenotype characterization of rat mesenchymal stromal cells. Cytotherapy 10, 243–253. https://doi.org/10.1080/14653240801950000
5. Fafián-Labora, J., Fernández-Pernas, P., Fuentes, I., De Toro, J., Oreiro, N., Sangiao-Alvarellos, S., Mateos, J., Arufe, M.C., 2015. Influence of age on rat bone-marrow mesenchymal stem cells potential. Sci Rep 5, 16765. https://doi.org/10.1038/srep16765
6. Liang, C.-C., Park, A.Y., Guan, J.-L., 2007. In vitro scratch assay: a convenient and inexpensive method for analysis of cell migration in vitro. Nat Protoc 2, 329–333. https://doi.org/10.1038/nprot.2007.30
7. Carpentier, G., Berndt, S., Ferratge, S., Rasband, W., Cuendet, M., Uzan, G., Albanese, P., 2020. Angiogenesis Analyzer for ImageJ — A comparative morphometric analysis of “Endothelial Tube Formation Assay” and “Fibrin Bead Assay.” Sci Rep 10, 11568. https://doi.org/10.1038/s41598-020-67289-8
